# Economic Value of Apple Pollination in the European Union, 2000 to 2024

**DOI:** 10.3390/insects17101038

**Published:** 2026-10-09

**Authors:** Janusz Majewski, Agnieszka Sobolewska

**Affiliations:** Department of International Economics and Agribusiness, Warsaw University of Life Sciences—SGGW, Nowoursynowska 166, 02-787 Warsaw, Poland; agnieszka_sobolewska@sggw.edu.pl

**Keywords:** apple orchards, pollination, insect pollination, pollination value, ecosystem service, economic value, economic value of insect pollination method, European Union

## Abstract

Insect pollination of crops is an important ecosystem service. Estimating the economic value of this service is a significant challenge. Apple orchards were selected for this study because apple trees are the main crop in the European Union that requires insect pollination to produce a crop. The aim of the study was to estimate the economic value of pollination as an ecosystem service in European Union countries from 2000 to 2024. The calculations used the economic value of production dependence on insect pollination method. The study assumed a 90% impact of insect pollination on apple yields and the average annual apple prices obtained by producers in individual EU countries. Data from the FAOSTAT database were used. The results indicate that between 2000 and 2024, the economic value of apple orchard pollination averaged 7 billion USD annually, with a range of 5.5 to 9 billion USD. The average pollination value of 1 hectare of apple orchard in the European Union increased from almost 9000 USD in 2000 to almost 14,000 USD in 2024. Over the years and within individual countries, the value of pollination varied significantly (from 0.2 to 95.4 thousand USD per ha). The main factors influencing differences in pollination value across EU countries were apple yields and prices received by producers.

## Simple Summary

Insect pollination of crops is an important ecosystem service. Apple trees are among the major crops that depend on insect pollination to produce a crop. The aim of this study was to estimate the economic value of pollination as an ecosystem service in European Union Member States over the period of 2000–2024. The calculations were based on the economic value of crop production attributable to insect pollination, which allows for estimating the value of crops produced as a result of insect-mediated pollination. The study assumed that insect pollination had a 90% impact on apple yields and producer apple prices. Data from the FAOSTAT (Food and Agriculture Organization of the United Nations) database were used. The results indicate that between 2000 and 2024, the economic value of pollination services in apple orchards averaged 7 billion USD annually, ranging from 5.5 to 9 billion USD. The average value of pollination for 1 hectare of apple orchard in the European Union increased from almost 9000 USD in 2000 to almost 14,000 USD in 2024. Over the study period and across individual countries, the value of pollination varied significantly (from 0.2 to 95.4 thousand USD per ha), reflecting differences in apple yields and prices received by producers.

## 1. Introduction

Ecosystems provide humans with many goods and services essential for their lives. The value of ecosystem services globally has been estimated at more than twice the global GDP [1,2] and is expected to grow in subsequent years [1,2]. Ecosystem services affect every human being. They are particularly important for agriculture [3,4,5,6,7,8], but they also impact other areas of human life [9,10,11,12,13]. Costanza et al. (1997) [14] identified 17 key ecosystem services. These can be divided into four types of environmental services: provisioning, regulating, cultural, and supporting [3,15,16].

Agriculture relies on a variety of services provided by the natural environment [17]. The development of agriculture worldwide, especially conventional agriculture focused on maximizing production, negatively impacts the environment. Agricultural intensification leads to reduced biodiversity in the natural environment [18,19] and a reduction in the value of ecosystem services [20]. To counteract this, a comprehensive approach to agriculture’s functions is necessary. The development of the concept of multifunctional [21,22,23] or sustainable agriculture [5] limits the negative effects of this activity. Agricultural changes should adopt the concept of the green economy [6]. This concept provides an interpretation of the operationalization of sustainable development, emphasizing the importance of well-being, social equality, ecological scarcity, and environmental risk [24]. The implementation of sustainable development includes the protection and use of insects for pollinating cross-pollinating crops [25,26,27,28].

One of the ecosystem services is plant pollination [14,26,29]. The pollination media of cross-pollinated plants are wind, water, and animals. Most cross-pollinated plant species are pollinated by animals [30,31]. It is estimated that nearly 350,000 animal species worldwide are involved in pollination. This number, in addition to insects, also includes over 1400 species of vertebrates, i.e., birds, mammals (mainly bats), and lizards that pollinate plants in warm regions of the world [32]. However, insects play a crucial role in plant pollination, constituting over 99.5% of pollinating animal species.

Pollination is an ecosystem service that ensures the reproduction of cultivated and wild plants [33,34,35,36], which supports the maintenance of biodiversity [18,33,37,38]. Globally, approximately 87% of plant species require animal pollination [30]. Insect pollination contributes to increased yields for approximately 1500 cultivated plant species, which constitute approximately 85% of crop species [36,39,40,41]. Of the 115 major crops worldwide, 87 species depend on insect pollination for their yields [41,42,43,44]. It is estimated that approximately 35% of the world’s food is obtained through insect pollination [35,41,45], and the area of entomophilous crops worldwide has tripled since the 1960s [46]. Insect pollination plays a particularly important role in the yields of fruits, oil plants, and some vegetables [41,47,48,49].

The economic value of crop pollination services worldwide has been estimated to range widely from 112 to 387 billion USD annually [14,39,50,51,52]. These estimates vary considerably due to the use of different methods and data from different years. Despite these differences, the economic value of crop pollination has been shown to increase over time, both globally and in the European Union [35,39,53] and nationally [47,54,55,56,57,58]. The increase in the value of crop pollination over time is also indicated by the studies of Hanley et al. [59] and Breeze et al. [60].

Valuing crop pollination is difficult because, in agricultural production, proper pollination is not the only or even a sufficient factor determining yields. Crop yields and quality also depend on factors such as weather conditions during growth and harvest, the occurrence of unexpected and severe weather events, the proper and timely application of agrotechnical practices, the variety of crops being cultivated, and the farmer’s knowledge. However, insect pollination differs from other agrotechnical practices in that it increases plants’ yield potential [61]. Other procedures performed by farmers are intended to utilize this potential. However, pollination alone does not guarantee yields.

The economic value of plant pollination can be estimated using various methods [36,39,59,60,62,63]. This is due to the complexity of factors influencing the quantity and quality of entomophilous plant production. The most commonly used method for estimating the value of pollination of cultivated entomophilous plants is the method based on the value of production dependence on pollination [59,64].

The most important crop requiring insect pollination in the European Union is the apple tree [65]. In 18 EU countries, apple cultivation is one of the three most important crops requiring pollination. The economic value of apple pollination accounted for approximately 16% of the total value of crop pollination in EU countries [53]. Furthermore, apples are among the most important fruits produced worldwide [66]. They account for the largest share of fruit production in EU countries [67]. Therefore, apple orchards were selected to study the economic value of pollination in European Union countries.

The aim of this paper is to estimate the value of the ecosystem service provided by insect pollination of apple orchards in European Union countries between 2000 and 2024. The method based on the economic value of production dependence on insect pollination was used to estimate the value of apple orchard pollination. Using the same method to estimate pollination values over time allows for inter-period and international comparisons and the identification of trends in the estimated values. This approach is unique in relation to the value of apple orchard pollination. The existing literature is dominated by publications reporting pollination values from single years, estimated using different methods, making it difficult or impossible to compare results.

## 2. Materials and Methods

The method of economic value production dependence on pollination was used to estimate the value of pollination services for apple orchards in European Union countries [36,39,60,62,68,69,70]. This is the most commonly used method in estimating the pollination value of crop plants [59,60]. To determine the value using this method, data on production volume, unit production price, and the dependence of yield on insect pollination are needed (Formula (1)).

EVP = Q · D · P,(1)

where

EVP—economic value of plant pollination by insects;
Q—annual production volume of plant (apples);
D—dependence of crops (apple orchards) on pollination by insects;
P—average annual purchase price of fruits (apples).

Data on the area of apple orchards, the volume of apple production, and the average purchase prices of apples in European Union countries for the years 2000–2024 were obtained from the Food and Agriculture Organization of the United Nations (FAOSTAT) database [71]. Annual food price inflation rates published by FAOSTAT [71] were used to calculate the value of pollination at constant 2024 prices.

Data regarding the impact of pollination on apple orchard yields were obtained from the literature. Research findings in this area vary widely [39,47,62,68,69,70,72,73,74,75]. Depending on the study, the impact of insect pollination on apple yields ranged from 0.1 to 1 [39]. This variation could be attributed to factors such as apple cultivars, the selection of cross-pollinating varieties, planting patterns, and weather conditions [76,77,78,79,80,81,82,83]. Nevertheless, the vast majority of apple cultivars require insect pollination to achieve high yields of good quality [39,60,84]. Apple pollination is considered the most critical ecosystem service for apple production. It was most commonly assumed that the impact of insect pollination on apple yields falls within the range of 0.8 to 1.0 [47,62,68,72,73,85]. Therefore, the studies assumed an apple yield dependency on insect pollination of 0.9. The degree of dependence of apple yields on insect pollination also reflects the possibility of self-pollination or wind-pollination of apple blossoms, although such instances are rare and typically result in low yields [25,83].

A diversity of pollinating insects has a positive effect on the quantity and quality of apple yields [86,87,88]. While the honey bee is the primary pollinator in orchards [89,90,91,92], other pollinating insects (solitary bees, bumblebees, hoverflies) improve pollination efficiency and increase apple yields [87,89,93,94,95,96,97,98,99,100]. The study considered the dependence of apple yields on the aggregate of pollinating insects, without distinguishing between individual species. The same level of dependence of apple yields on insect pollination was assumed for all the countries studied. The study included countries that were members of the European Union in 2024—27 countries in total—regardless of their year of accession. This enables a comparison of the value of apple orchard pollination across the EU over time.

## 3. Results

### 3.1. Changes in Apple Production in European Union Countries from 2000 to 2024

**Figure 1:**
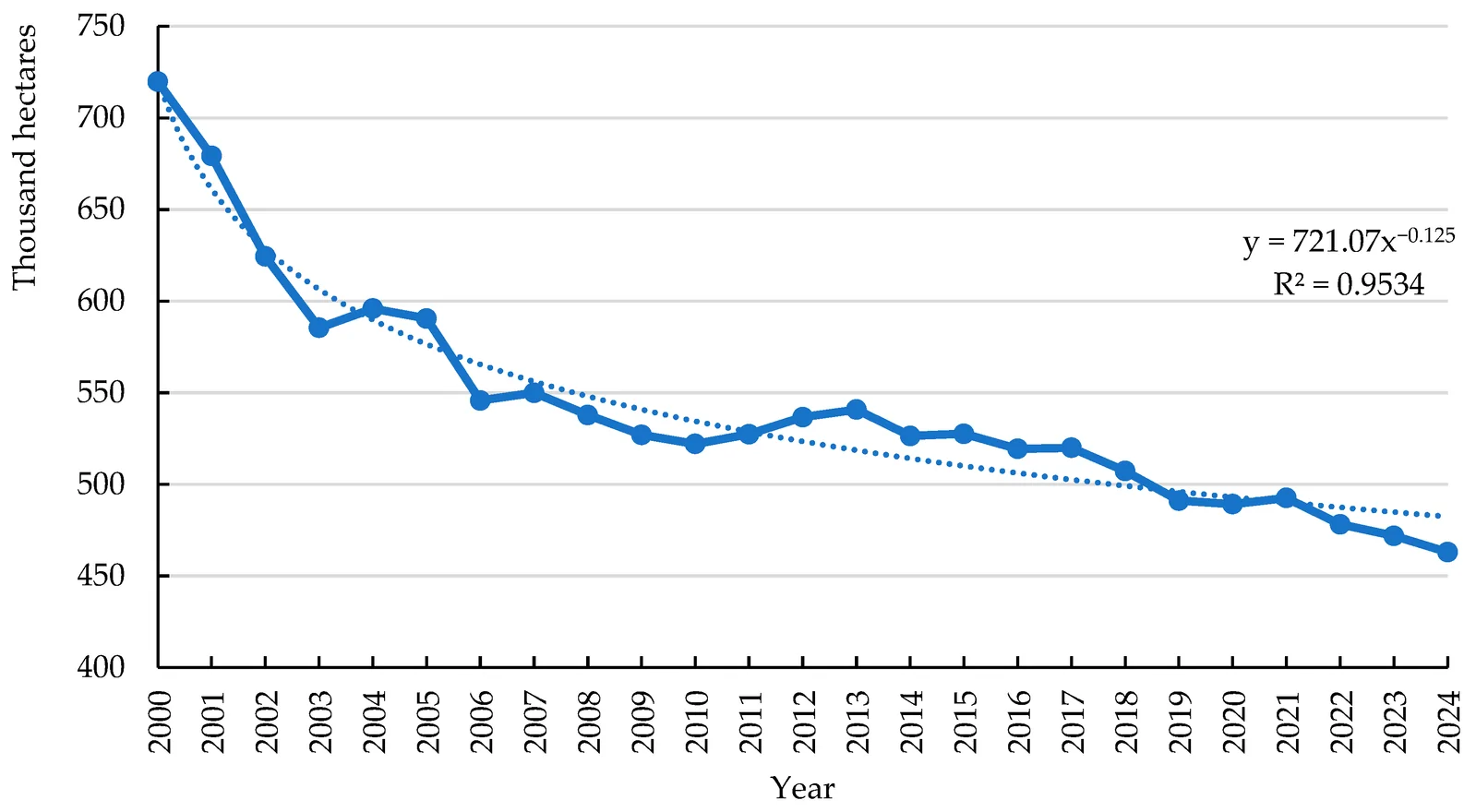
Area of apple orchards in the European Union in 2000–2024, in thousand ha.

Apples are one of the most important fruits cultivated in all European Union countries [66,71,101,102]. In terms of apple orchard area, the European Union ranks second only to China [103,104]. The area under apple cultivation in the EU systematically decreased between 2000 and 2024 (Figure 1).

Between 2000 and 2024, the area of apple orchards in the EU decreased by approximately 35%. The largest declines in apple orchard area in these years occurred in Germany and the Czech Republic (approx. 74% each), Bulgaria and Lithuania (approx. 73% each), and in the EU countries with the smallest apple orchard areas (Cyprus, Estonia, Finland, Ireland, Luxembourg, Malta) (Table A1). The highest rate of decline occurred in 2000–2003. During this period, the area of apple orchards decreased by almost 20%. This was mainly due to a decrease in the area under apple trees in Germany (by 75%) and, to a lesser extent, declines in Bulgaria (by 64%), the Czechia (by 54%), and Lithuania (by 38%). In the following years (2004–2024), the rate of decline in the area of apple orchards in the EU slowed (Figure 1) to approximately 5000 hectares per year on average.

**Figure 2:**
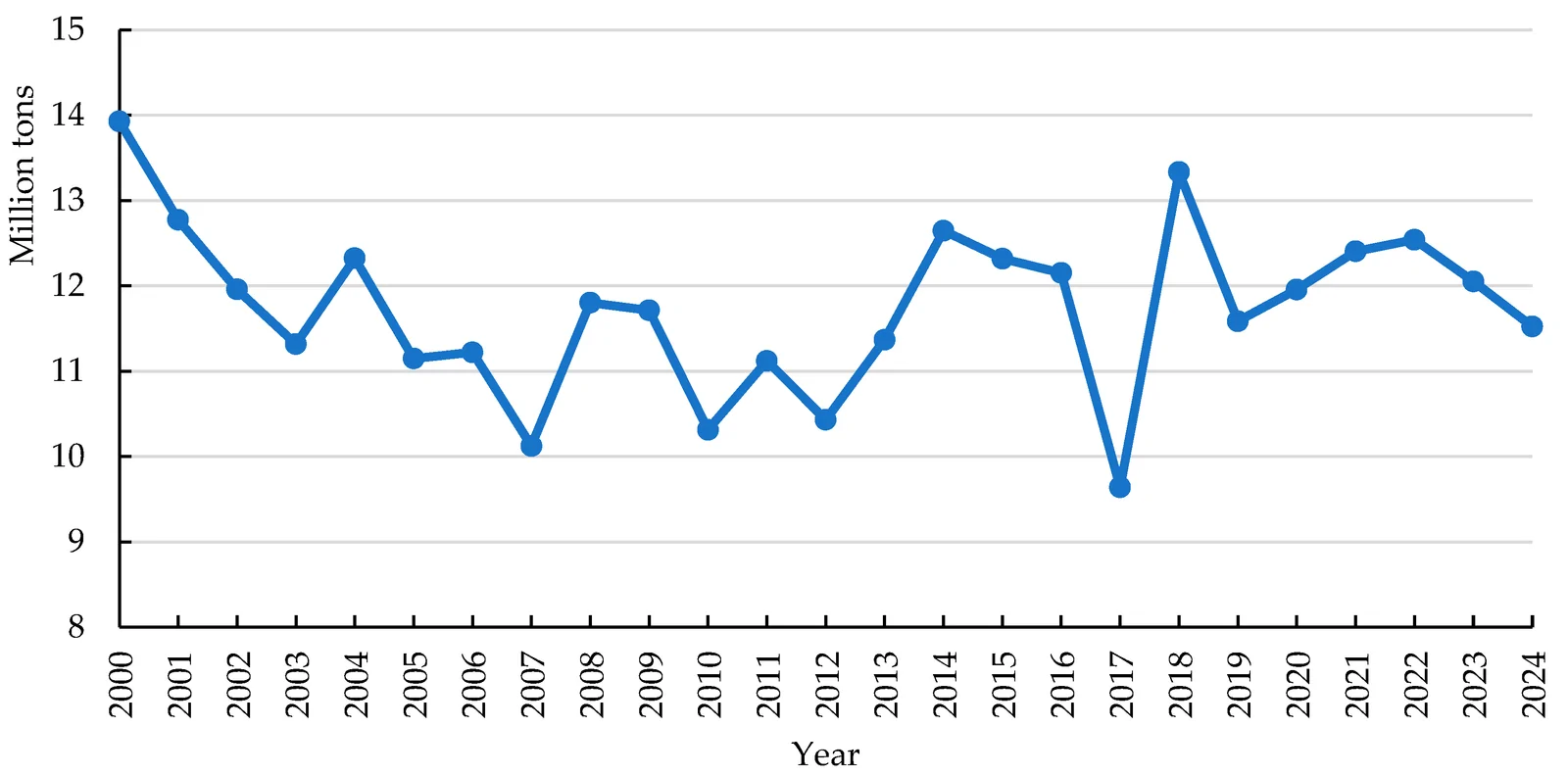
Apple production in the European Union countries in 2000–2024, in million tons.

**Figure 3:**
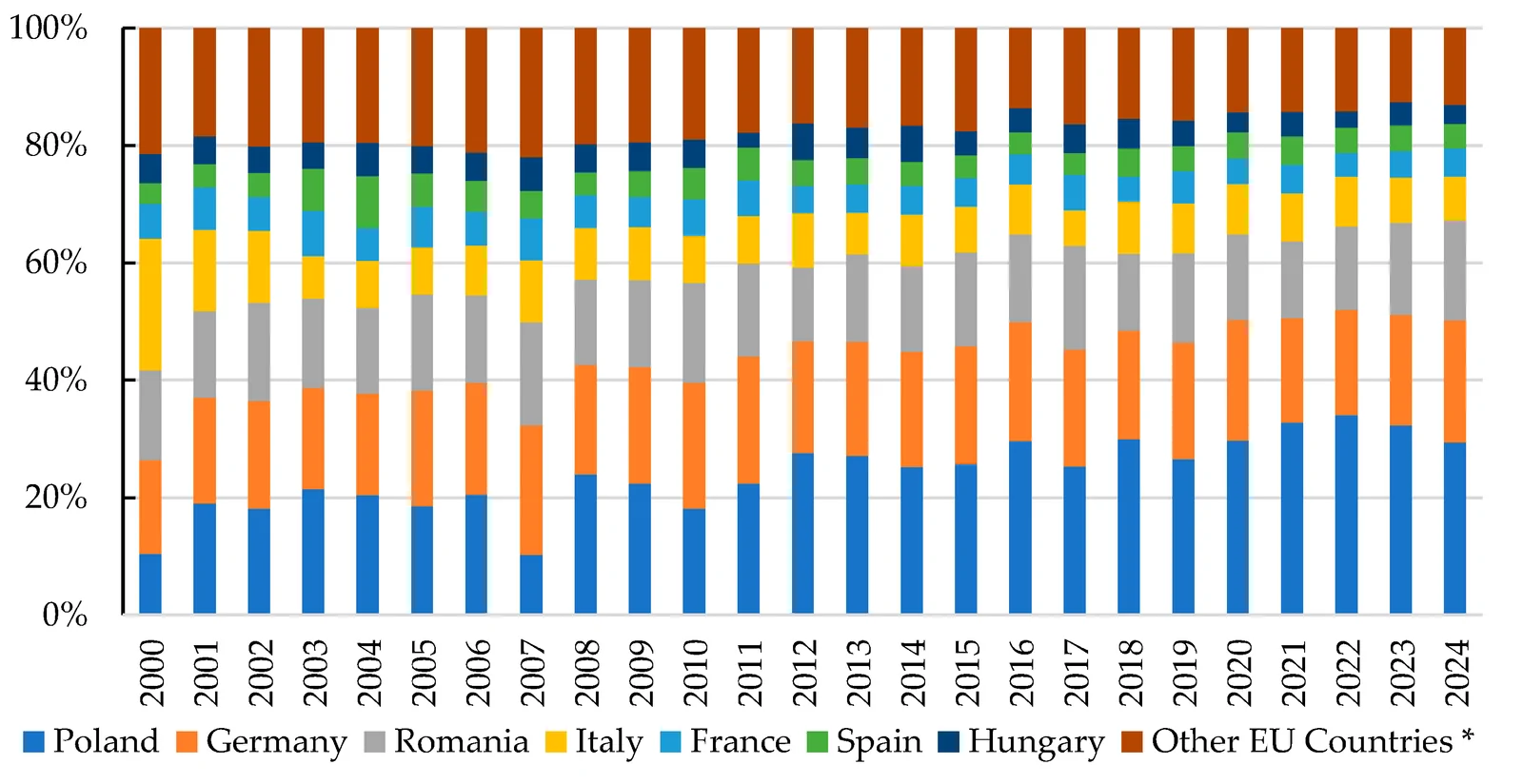
Diversity of apple yields in European Union countries. *—the remaining 20 EU countries.

Despite the decline in apple orchard area, apple production in the EU did not decline between 2000 and 2024. However, it was characterized by significant, stationary fluctuations, from less than 10 million tons in 2017 to almost 14 million tons in 2000 (Figure 2). The country with the highest growth in apple production was Poland [69,76,79], which led to an increase in its share of EU apple production from 10.4% in 2000 to an average of over 30% in 2020–2024 (Table A2)—among other significant apple producers in the EU, Italy and France had a relatively stable share in production volume during the period under review, amounting to 16.0–20.8% (Italy) and 12.5–17.6% (France), respectively, of EU production. In Germany, the share of apple production decreased from 22.5% in 2000 to an average of around 8% of EU production in 2020–2024 (Table A2). The four main apple producers in the EU (France, Germany, Italy, and Poland) accounted for 60–75% of EU apple production. The structure of apple production in EU countries is shown in Figure 3.

**Figure 4:**
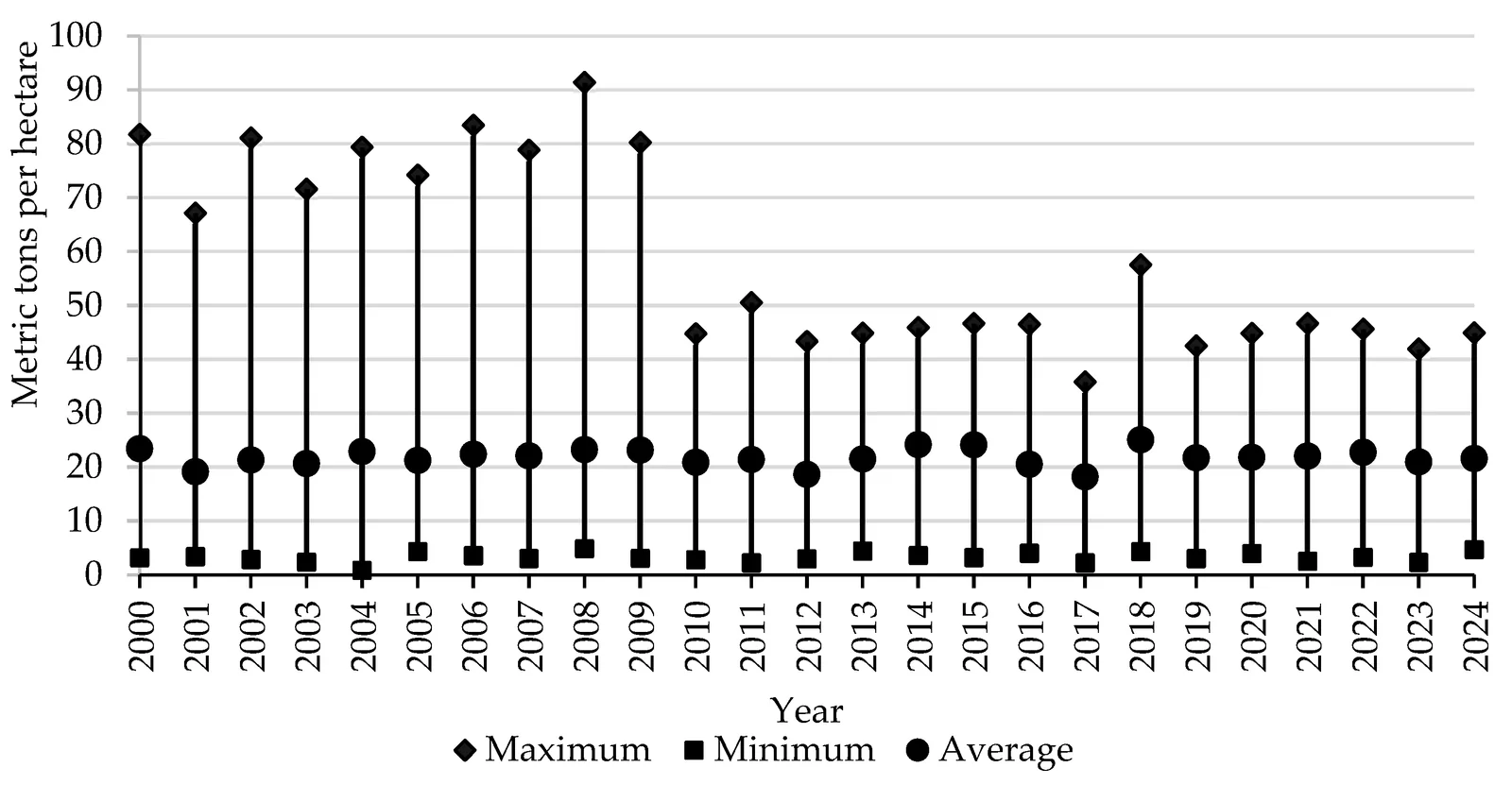
Diversity of apple yields in European Union countries in 2000–2024, in t per ha.

There are many factors influencing apple yields, including climatic, economic, technical, and social factors [105,106,107,108]. Since 2000, apple production in EU countries has intensified. There was a gradual shift from traditional orchards to high-yield dwarf orchards. However, the fruit-growing sector across the EU remains diverse in this regard. Apple production in the EU is subject to weather-related risks, primarily spring frosts, droughts, and hailstorms. While innovations in fruit growing help mitigate these risks, they do not eliminate them [66,104,109,110,111]. Average apple yields across European Union countries from 2000 to 2024 showed a systematic increase (Figure 4).

During the analyzed period, there was significant variation in yields across EU countries. The lowest average yields were achieved by farmers in Lithuania and Latvia, at 4–32% of the EU average (Table A3). This could be due to more severe climatic conditions in these regions than in other EU countries. The highest average yields in 2000–2010 were achieved in Austria. In subsequent years, the countries with the highest average apple yields were France, Belgium, the Netherlands, Austria, and Italy (Table A3). There has been a noticeable reduction in variation in average yields across the countries studied over time, which may indicate improvements in production technology in low-yield countries. This is particularly evident in Poland, where average yields increased from approximately 45% of the EU average in 2000 to levels close to the EU average in 2020–2024 (Table A3).

**Figure 5:**
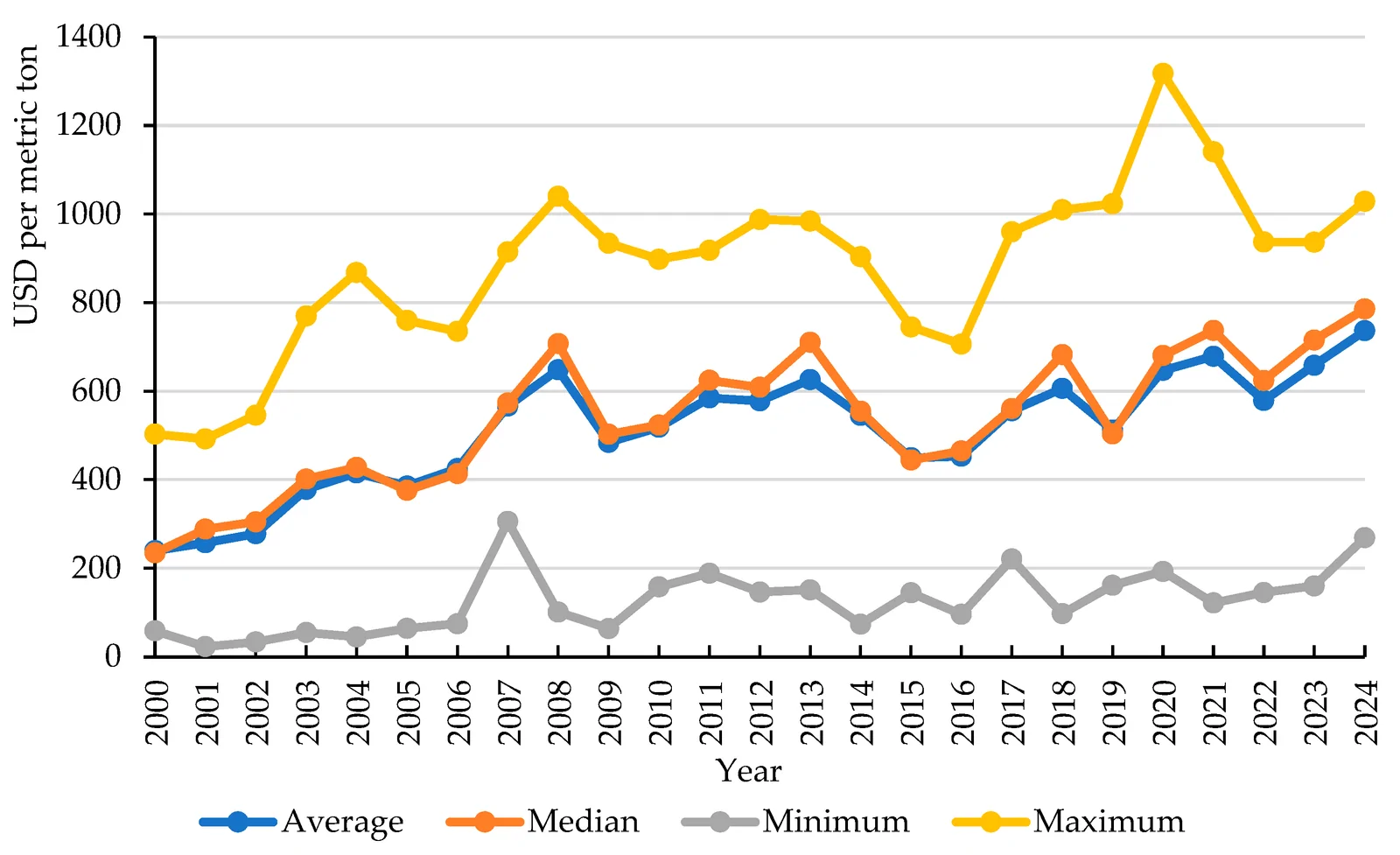
Differences in apple prices in European Union countries according to producer prices (current prices), in USD per t.

The selling price of apples is the basis for the income potential of apple growers. In our study, apple prices were an important element in estimating the value of pollination in apple orchards. Between 2000 and 2024, current producer prices increased over time, with significant random fluctuations. Average current prices increased from nearly 240 USD per ton of apples in 2000 to 736 USD per ton in 2024 (Figure 5).

Within the European Union, there was significant variation in average apple prices. Differences in apple producer prices in EU countries ranged from approximately 450 USD per ton in 2000 to over 1100 USD per ton in 2020. Apple producers typically achieved the lowest average prices in countries with low purchasing power, and the highest in Western and Southern European countries with low apple supply.

### 3.2. Pollination Economic Value for European Union Apple Orchards

To estimate the value of pollination in apple orchards in the European Union, the economic production value dependence on pollination method, which is used to estimate the value of pollination of crop plants [51,66,71,72], was applied. The impact of pollination on the obtained yield was assumed to be 90%. To estimate the economic value of pollination, average apple prices in European Union countries, based on producer prices, were considered (Figure 5). To compare results over time, pollination values were adjusted to constant 2024 prices using food price inflation indicators for each country, as published by FAOSTAT [71].

**Figure 6:**
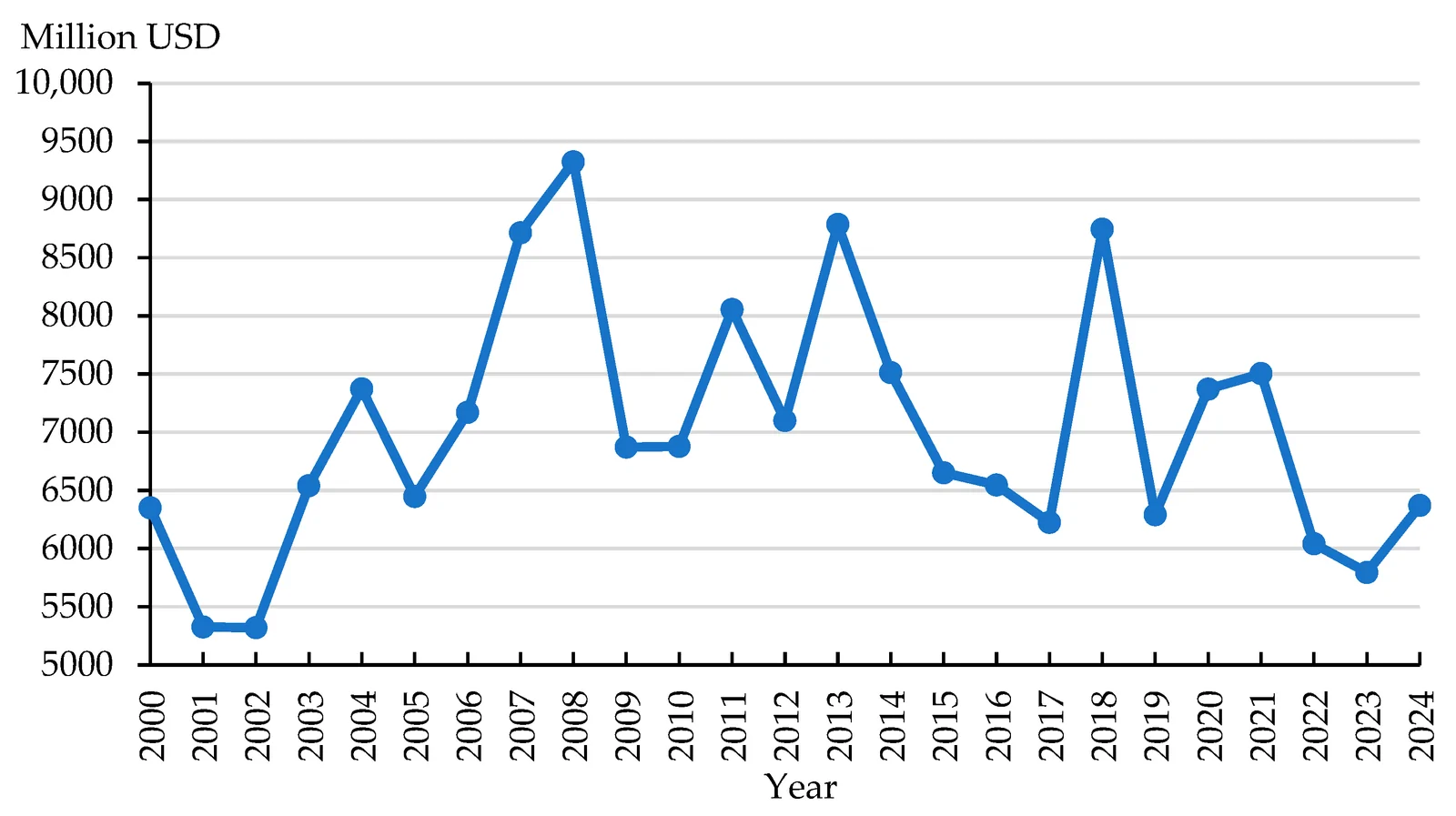
The value of pollination of apple orchards in the European Union in 2000–2024 (constant 2024 prices), in million USD.

The value of pollination of apple orchards in the European Union for 2000–2024, estimated using the method of production value dependence on pollination, ranged from 5.3 billion USD in 2002 to 9.3 billion USD in 2008 (Figure 6). There is significant variation in the value of pollination across subsequent years, which may result from changes in both apple production volume and the prices fruit growers receive for apples.

**Figure 7:**
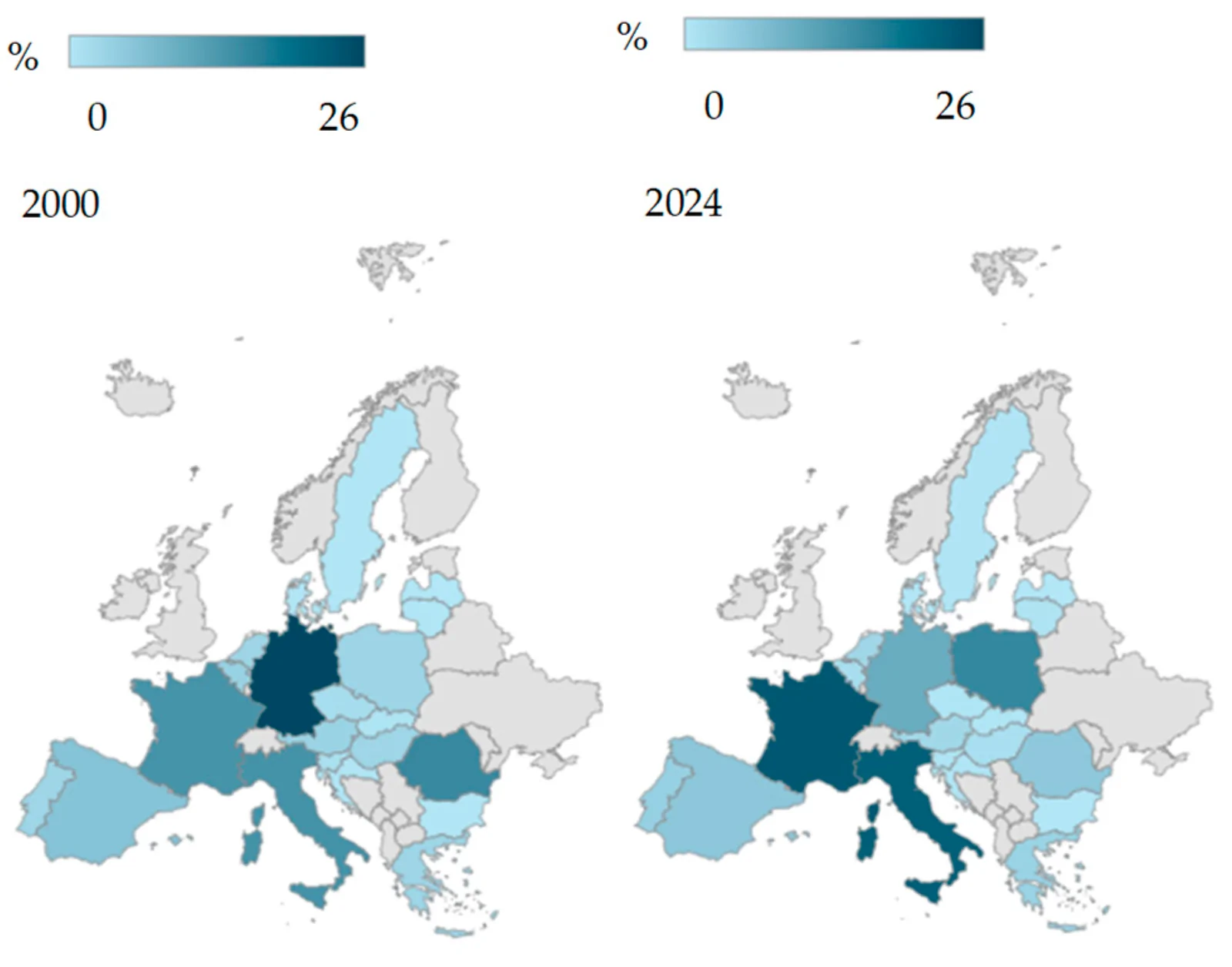
Structure of the economic value of apple orchard pollination in EU countries in 2000 and 2024.

The share of the value of apple pollination services provided by insects in the EU for the four main apple producers (France, Germany, Italy, and Poland) ranged from 55.9% in 2000 to 70.8% in 2024 (Figure 7, Table A4). This increase was mainly due to higher apple orchard pollination value in Poland, driven by higher apple production and average prices received by producers. This resulted in Poland’s share of the value of apple pollination in the EU increasing from less than 3% in 2000 to 15.6% in 2024 (Table A4). However, although Poland produced the most apples in the EU in 2024, higher pollination values were recorded for France and Italy (23.3% and 22.2% of the EU apple pollination value, respectively), reflecting significantly higher prices for apple producers in these countries.

**Figure 8:**
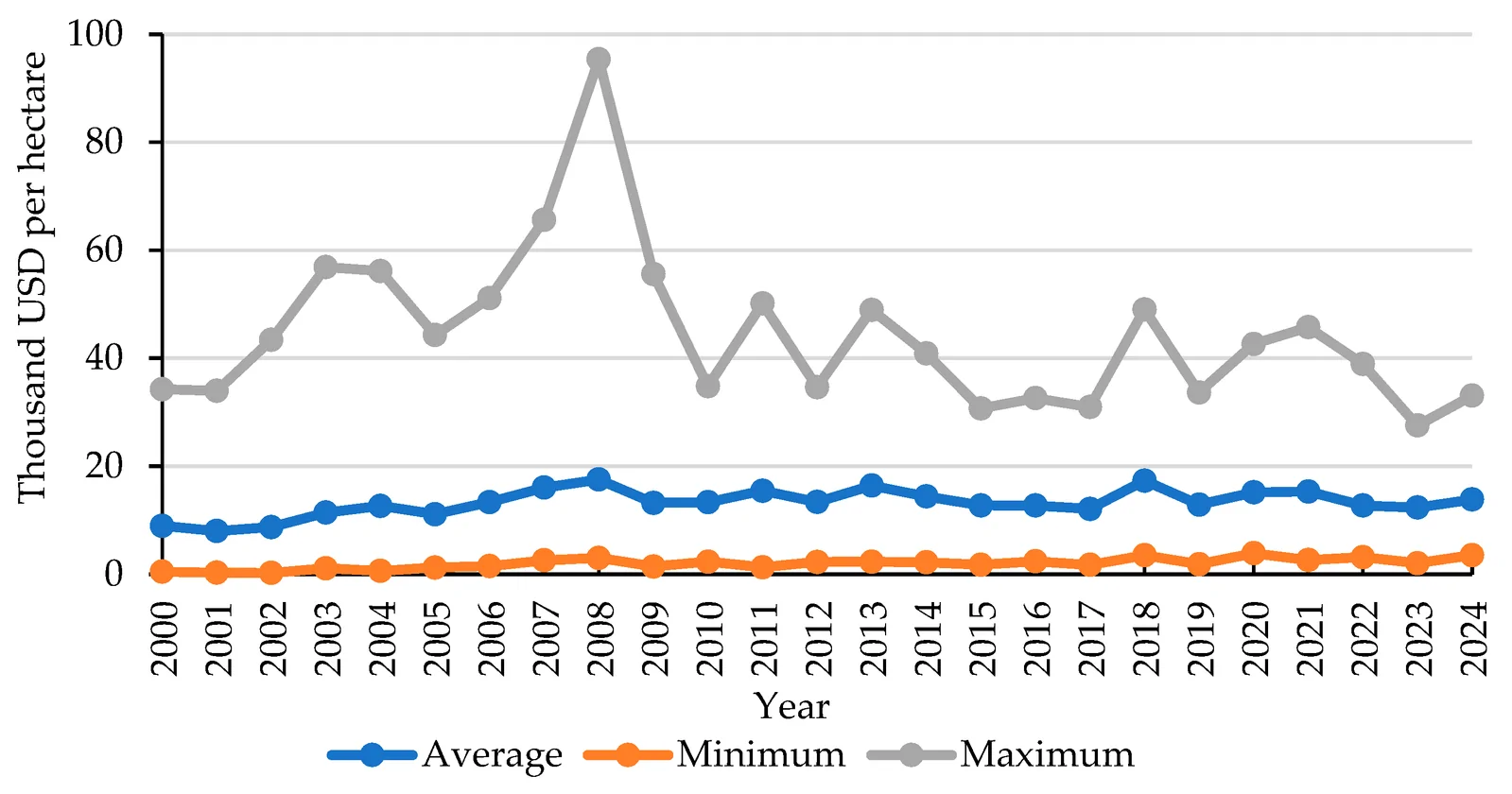
Differences in the pollination value of 1 ha of apple orchard in EU countries in 2000–2024 (constant 2024 prices), in thousand USD per ha.

The pollination value estimated for countries or regions is largely based on production scale. This means that the highest pollination value for apple orchards in the EU was achieved in countries with the highest production scale. To compare the pollination value of apple orchards in individual EU countries, the production value per crop unit (hectare of apple orchard) was estimated (Figure 8).

The average pollination value per hectare of apple orchards in the EU in 2000–2024 was 13.24 thousand USD, ranging from 8 thousand USD in 2001 to 17.55 thousand USD in 2008. The lowest pollination values were obtained in Lithuania and Latvia, ranging from 0.2 thousand to 6.7 thousand USD per hectare (Table A5). The highest average pollination values were observed in apple orchards in Austria, the Netherlands, and France. The highest pollination values per hectare of apple orchard in individual years ranged from 30.0 thousand to 95.4 thousand USD (Table A5). This indicates a substantial variation in pollination values across countries, driven by significant differences in yields and prices received by apple producers.

**Figure 9:**
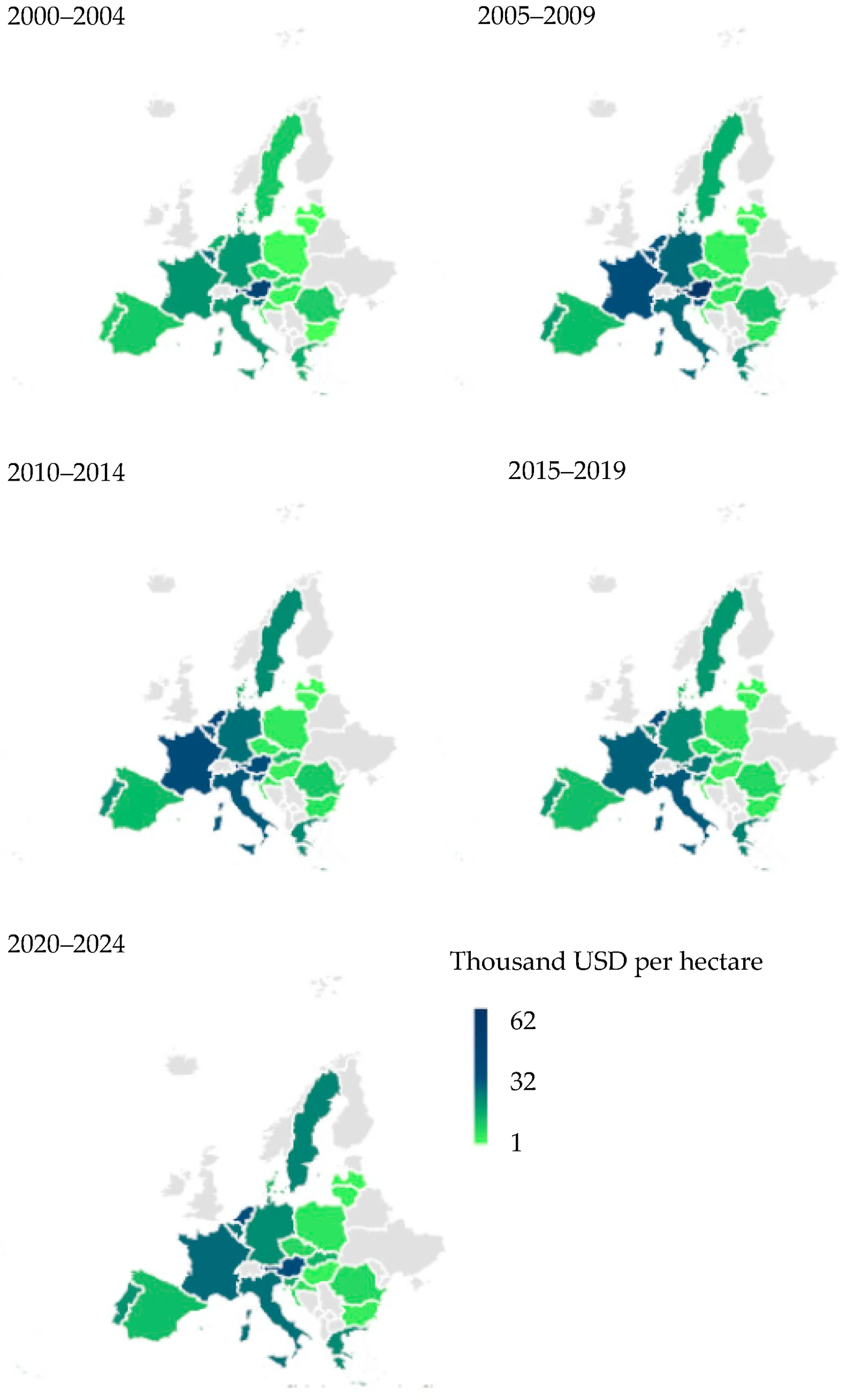
Changes in the economic value of apple orchard pollination in European Union countries (2000–2024).

To determine changes in the average pollination value per hectare of apple orchard in EU countries, average values were calculated for successive five-year periods (Figure 9). This approach helped mitigate the impact of outliers due to weather and climate on the analyzed data. The results indicate a steady increase in the pollination value per hectare of apple orchard across the countries studied. Significant regional variation in apple pollination values persisted throughout the study period.

## 4. Discussion

This study adds to the limited body of research on the economic value of insect pollination of apple orchards, treated as an ecosystem service, across European Union countries over time. The economic value of production dependence on pollination method was used to estimate this value. The economic value of insect pollination of apple orchards in the European Union between 2000 and 2024, expressed in constant 2024 prices, averaged 7 billion USD annually, with fluctuations ranging from over 5 billion USD to nearly 9.5 billion USD. The size of the apple harvest influenced changes in the value of pollination during the study period each year, the prices obtained by producers, and changes in the spatial structure of apple production by EU country.

Even though apples are considered the most important crop in the European Union and require insect pollination [57], there is a lack of research on the economic value of pollination for this crop. Previous studies on the pollination of apple orchards by insects have focused mainly on determining their importance for apple yields [72,73,112,113]; the species of insects pollinating apple trees [76,80,87,90,93,94,95,96,97,114,115,116], especially honeybees [81,112,117]; the possibility of artificial pollination of plants by humans [118] or machines [119]; and the impact of climate change on pollinating insects [78,120]. Research results on the economic value of apple pollination as an ecosystem service are rare.

The global value of apple orchard pollination was estimated at 15.7 billion EUR in 2005 [69]. The economic value of apple orchard pollination has also been estimated for the USA [47,62], France [121], Great Britain [122,123], and China [58]. However, most of these studies are limited to estimating the pollination value for a single period and cannot indicate the direction of change over time. In our study, we made inter-period and inter-country comparisons. We demonstrated changes in the importance of individual EU countries in the overall value of apple orchard pollination. During the period under review, Poland’s share in the economic value of apple orchard pollination in the EU increased from less than 3% in 2000 to approximately 15% in 2024. This increase resulted, on the one hand, from higher apple production in Poland and, on the other hand, from lower apple production in Germany and, to a lesser extent, in Austria, Belgium, Czechia, and the Netherlands. In addition, the increase in Poland’s share of the total value of apple pollination in the EU was driven by an increase in average producer prices from 59 USD per ton in 2000, i.e., approximately 25% of the average producer price for apples in the EU, to 326 USD per ton in 2024, i.e., 44% of the average producer price for apples in the EU.

To compare the economic value of pollination in apple orchards across European Union countries, the value was estimated per unit area of apple orchards. Such calculations enable comparisons regardless of the scale of production. In EU countries, the economic value of pollination of 1 hectare of apple orchards showed an upward trend, with significant variation between countries, from 0.22 thousand USD in Lithuania in 2002 to 95.37 thousand USD in Austria in 2008. Such a large difference resulted from yields and producer prices in Austria in 2008 being over 30 times higher than in Lithuania in 2002. In general, throughout the entire period under study, the lowest values of apple pollination were obtained for Lithuania and Latvia, which were mainly due to low yields obtained by apple growers in these countries (apple yields in these countries constituted, on average, about 20% of the average yield in the EU). In turn, the highest pollination values per hectare of apple orchard were observed in countries with high apple yields, i.e., Austria, France, and the Netherlands.

The significant variation in the economic value of apple orchard pollination may also be due to differing natural conditions across European Union countries. Insect pollination of apple orchards increases the production potential of orchards. However, this potential can be limited or even destroyed by extreme natural phenomena, such as frosts during apple blossom or fruit formation, drought, or hailstorms. Furthermore, yield potential can be limited by the orchard owner, for example, through untimely agronomic practices or improper use of plant protection products. These factors cause significant variability in the economic value of apple orchard pollination at both the national and local levels.

It should be emphasized that the calculated pollination values for apple orchards were influenced by the underlying assumptions and the methodology employed. When the replacement cost method—assuming the replacement of insect pollinators with human labor—was applied, the resulting pollination value was 50% higher than that obtained using the method based on the value of production dependent on insect pollination [124]. Conversely, when using the method based on the cost of renting honeybee colonies for apple pollination, the value of pollinating one hectare of apple orchard equals the rental cost of 4 to 5 honeybee colonies [117]. With this method, the cost can be determined by the market (through negotiations between the orchard owner and the beekeeper regarding the rental price) or by the opportunity cost (i.e., the difference between the honey yield the rented colonies could have obtained from nectar-rich crops blooming concurrently with the apple trees—such as rapeseed—and the actual yield obtained in the apple orchard). The average rental cost of a honey bee colony for apple orchard pollination in 2024 was approximately USD 58 [125], with significant variation depending on the country and the bee colony’s strength [126,127]. Comparing crop pollination values derived from different methodologies could be an interesting subject for future research.

Further research on the economic value of insect pollination in apple orchards could assess the value of pollination by individual pollinator species. Studies could also be undertaken to compare the pollination values of orchards obtained using different methods and identify the reasons for these differences. This would allow for a broader perspective on the problem of estimating the economic value of pollination as an ecosystem service.

## Data Availability

Data are available in a publicly accessible repository.

## Appendix A

**Table A1:** The structure of apple orchard areas in the European Union countries from 2000 to 2024.

Country	2000	2001	2002	2003	2004	2005	2006	2007	2008	2009	2010	2011	2012	2013	2014	2015	2016	2017	2018	2019	2020	2021	2022	2023	2024
Total, thousand hectares	719.8	679.3	624.4	585.4	595.9	590.5	545.8	550.0	537.8	527.0	522.0	527.3	536.7	540.8	526.4	527.5	519.4	520.0	507.3	491.1	489.2	492.5	478.1	471.9	462.9
Share of apple orchard area in the EU, in %																									
Austria	0.83	0.90	0.94	1.01	1.02	1.03	1.12	1.10	1.12	1.15	1.16	1.15	1.13	1.29	1.28	1.25	1.28	1.28	1.33	1.34	1.31	1.29	1.32	1.32	1.29
Belgium	1.24	1.30	1.38	1.43	1.39	1.51	1.58	1.55	1.51	1.46	1.48	1.47	1.32	1.31	1.35	1.30	1.25	1.19	1.18	1.18	1.12	1.09	1.09	1.04	0.97
Bulgaria	1.80	1.45	0.96	0.80	0.76	0.53	0.55	0.64	0.60	0.82	1.00	0.93	0.86	0.89	0.75	0.90	0.79	0.76	0.78	0.84	0.73	0.77	0.78	0.75	0.76
Croatia	0.58	0.71	0.86	1.06	0.88	0.95	1.07	1.09	1.19	1.24	1.26	1.24	1.11	0.99	1.13	1.09	1.13	0.93	0.93	1.01	0.89	0.89	0.76	0.77	0.83
Czechia	2.92	2.65	1.48	1.59	1.59	1.62	1.70	1.65	1.68	1.71	1.73	1.76	1.75	1.66	1.70	1.58	1.44	1.41	1.43	1.49	1.47	1.44	1.47	1.37	1.18
Denmark	0.23	0.26	0.25	0.28	0.27	0.27	0.28	0.27	0.28	0.28	0.31	0.29	0.25	0.26	0.26	0.26	0.26	0.25	0.28	0.28	0.28	0.28	0.32	0.31	0.32
France	7.37	7.42	7.92	8.31	7.95	7.91	8.04	7.86	7.82	7.82	7.84	7.53	7.21	6.95	9.53	9.41	9.66	9.68	9.96	10.26	11.18	11.01	11.30	11.40	11.40
Germany	17.36	10.31	8.76	5.32	5.43	5.48	5.96	5.77	5.91	6.04	6.10	5.99	5.90	5.85	5.98	5.95	6.03	6.52	6.70	6.92	6.95	6.90	6.92	7.02	7.15
Greece	1.96	2.04	2.19	2.61	2.25	2.26	2.44	2.40	2.50	2.57	2.58	2.53	2.48	2.44	1.86	1.86	1.86	1.79	2.04	2.00	2.94	2.09	2.23	2.07	1.97
Hungary	4.79	8.42	5.74	7.43	7.26	7.12	7.17	7.36	8.01	6.95	6.52	6.28	5.97	6.17	6.32	6.22	6.25	6.19	6.28	6.31	5.31	5.08	4.98	4.83	4.87
Italy	8.69	9.22	9.69	9.72	9.66	9.68	10.47	10.19	10.97	11.09	11.09	10.78	10.19	10.22	10.40	10.37	10.16	10.31	11.32	11.20	11.22	11.06	11.24	11.46	11.53
Latvia	1.13	1.20	1.32	1.41	1.40	1.44	1.73	1.34	0.96	0.79	0.62	0.64	0.58	0.51	0.50	0.46	0.47	0.64	0.63	0.70	0.72	0.65	0.64	0.70	0.67
Lithuania	4.31	4.61	4.47	3.29	3.24	3.52	2.72	2.42	2.17	2.19	2.32	2.40	2.68	2.63	2.63	2.51	2.36	2.38	2.00	2.07	2.15	2.07	2.07	1.91	1.81
Netherlands	1.78	1.73	1.79	1.76	1.71	1.65	1.75	1.71	1.73	1.73	1.66	1.57	1.48	1.46	1.49	1.44	1.41	1.34	1.30	1.31	1.27	1.21	1.23	1.17	1.13
Poland	22.94	24.50	26.98	27.21	29.40	28.73	29.68	31.93	31.97	32.94	32.65	34.80	36.28	35.77	33.50	34.20	34.12	33.92	32.76	31.69	31.20	32.87	31.77	31.79	31.97
Portugal	2.93	3.00	3.16	3.26	3.02	2.85	2.88	2.65	2.56	2.38	2.38	2.38	2.40	2.53	2.63	2.65	2.77	2.84	2.87	2.91	2.93	2.83	2.87	2.95	2.95
Romania	9.87	10.75	11.60	12.23	12.31	13.83	10.86	10.73	10.17	9.99	10.80	10.00	10.32	11.15	10.66	10.59	10.69	10.69	10.63	10.74	10.70	10.93	11.31	11.51	11.58
Slovakia	0.42	0.61	0.75	0.60	0.55	0.54	0.61	0.59	0.64	0.52	0.51	0.44	0.54	0.52	0.49	0.45	0.44	0.42	0.42	0.42	0.37	0.33	0.32	0.33	0.33
Slovenia	0.43	0.45	0.50	0.53	0.52	0.52	0.57	0.52	0.53	0.52	0.53	0.52	0.50	0.49	0.48	0.47	0.47	0.45	0.46	0.46	0.44	0.42	0.42	0.42	0.41
Spain	6.77	6.23	6.92	7.86	7.08	6.60	6.93	6.71	6.20	6.21	6.09	5.97	5.73	5.70	5.84	5.82	5.94	5.88	5.90	6.04	6.03	5.98	6.12	6.02	6.04
Sweden	0.20	0.21	0.21	0.23	0.23	0.24	0.29	0.25	0.26	0.28	0.29	0.25	0.28	0.26	0.25	0.25	0.30	0.27	0.28	0.31	0.29	0.30	0.30	0.31	0.31
Others *	1.45	2.04	2.13	2.05	2.08	1.72	1.60	1.26	1.21	1.32	1.08	1.08	1.03	0.96	0.97	0.94	0.89	0.87	0.51	0.52	0.51	0.52	0.54	0.55	0.54
Total	100.0	100.0	100.0	100.0	100.0	100.0	100.0	100.0	100.0	100.0	100.0	100.0	100.0	100.0	100.0	100.0	100.0	100.0	100.0	100.0	100.0	100.0	100.0	100.0	100.0

*—other EU member states in 2024, i.e., Cyprus, Estonia, Finland, Ireland, Luxembourg, and Malta.

**Table A2:** The structure of apple production in the European Union countries from 2000 to 2024.

Country	2000	2001	2002	2003	2004	2005	2006	2007	2008	2009	2010	2011	2012	2013	2014	2015	2016	2017	2018	2019	2020	2021	2022	2023	2024
Total, million tons	13.93	12.77	11.96	11.32	12.32	11.15	11.22	10.12	11.80	11.71	10.31	11.12	10.43	11.37	12.65	12.32	12.15	9.64	13.33	11.59	11.96	12.41	12.54	12.05	11.52
Share of EU apple production, in %																									
Austria	3.52	3.21	4.00	3.73	3.93	4.06	4.54	4.72	4.67	4.15	2.63	2.72	2.51	2.06	2.45	2.33	0.84	1.92	2.91	2.06	2.16	1.66	2.08	1.59	1.52
Belgium	3.92	2.64	2.92	2.82	2.89	2.91	3.19	3.54	2.84	2.65	3.34	2.05	2.11	2.01	2.52	2.31	1.96	0.89	1.73	2.09	1.41	2.01	1.90	1.68	1.39
Bulgaria	0.64	0.33	0.22	0.34	0.32	0.23	0.23	0.26	0.20	0.30	0.42	0.36	0.30	0.48	0.43	0.47	0.37	0.47	0.38	0.38	0.32	0.35	0.37	0.29	0.32
Croatia	0.46	0.18	0.37	0.41	0.51	0.51	0.51	0.62	0.49	0.63	0.86	0.90	0.36	1.07	0.76	0.83	0.37	0.59	0.68	0.59	0.53	0.48	0.37	0.51	0.59
Czechia	2.44	1.73	1.39	1.24	1.19	1.20	1.22	1.14	1.31	1.21	0.97	0.76	1.14	1.07	1.01	1.26	1.03	1.09	1.14	0.86	0.97	0.93	1.05	0.84	0.33
Denmark	0.18	0.16	0.16	0.28	0.29	0.32	0.28	0.32	0.25	0.23	0.23	0.23	0.18	0.27	0.28	0.29	0.30	0.21	0.24	0.22	0.33	0.25	0.17	0.11	0.16
France	15.29	14.73	16.69	15.21	14.61	16.41	14.97	17.60	14.42	14.77	16.98	15.85	12.52	14.85	14.61	15.98	15.00	17.59	13.05	15.14	14.58	13.16	14.24	15.72	17.04
Germany	22.52	13.93	12.30	7.23	7.95	8.00	8.44	10.57	8.87	9.14	8.10	8.08	9.32	7.07	8.82	7.90	8.50	6.19	8.99	8.56	8.56	8.10	8.54	7.81	7.57
Greece	2.26	1.94	2.35	1.83	2.23	2.24	2.57	2.48	2.20	2.29	2.65	2.46	2.55	2.23	1.99	2.17	2.30	2.63	2.14	2.39	2.54	2.31	2.31	2.01	2.28
Hungary	4.99	4.74	4.41	4.48	5.68	4.58	4.79	5.79	4.82	4.91	4.82	2.63	6.24	5.21	6.17	4.15	4.09	4.91	5.09	4.30	3.33	4.15	2.79	4.03	3.27
Italy	16.02	18.00	18.39	17.26	17.34	19.66	18.99	22.03	18.73	19.85	21.38	21.68	19.09	19.50	19.56	20.08	20.21	19.93	18.50	19.88	20.59	17.83	17.99	18.82	20.81
Latvia	0.25	0.28	0.42	0.32	0.06	0.34	0.30	0.30	0.24	0.11	0.10	0.07	0.09	0.13	0.08	0.06	0.08	0.08	0.10	0.09	0.12	0.07	0.08	0.06	0.13
Lithuania	0.71	1.18	0.67	0.86	0.28	0.87	0.89	0.40	0.63	0.45	0.33	0.44	0.70	0.55	0.48	0.60	0.54	0.91	0.61	0.28	0.43	0.29	0.41	0.32	0.53
Netherlands	3.31	3.19	2.96	3.17	3.54	3.22	3.25	3.86	3.18	3.47	3.24	3.76	2.69	2.76	2.79	2.73	2.61	2.36	2.02	2.36	1.84	1.97	1.88	1.64	1.68
Poland	10.41	19.05	18.13	21.45	20.46	18.61	20.54	10.27	23.98	22.42	18.21	22.42	27.59	27.14	25.27	25.72	29.66	25.33	30.00	26.59	29.73	32.79	34.01	32.30	29.37
Portugal	1.65	2.02	2.51	2.52	2.23	2.25	2.29	2.42	2.01	2.25	2.06	2.22	2.12	2.53	2.16	2.64	1.99	3.42	1.98	3.20	2.39	2.97	2.32	2.42	2.72
Romania	3.52	3.97	4.11	7.17	8.91	5.72	5.26	4.70	3.89	4.42	5.36	5.58	4.44	4.52	4.06	3.86	3.85	3.62	4.76	4.25	4.49	4.79	4.33	4.43	4.23
Slovakia	0.59	0.39	0.43	0.54	0.34	0.42	0.27	0.18	0.35	0.32	0.33	0.28	0.43	0.40	0.38	0.38	0.17	0.34	0.33	0.30	0.24	0.24	0.25	0.22	0.29
Slovenia	0.93	0.61	1.18	0.90	1.14	0.95	1.06	1.13	0.87	0.82	1.14	0.95	0.53	0.81	0.79	0.93	0.49	0.83	0.65	0.47	0.55	0.19	0.39	0.25	0.44
Spain	5.84	7.18	5.81	7.79	5.61	6.94	5.80	7.12	5.61	5.14	6.26	6.03	4.61	4.81	4.91	4.82	5.11	6.09	4.22	5.51	4.37	4.96	3.96	4.38	4.73
Sweden	0.16	0.16	0.15	0.19	0.15	0.16	0.21	0.21	0.19	0.18	0.23	0.19	0.22	0.24	0.19	0.21	0.22	0.23	0.23	0.19	0.25	0.26	0.25	0.27	0.27
Others *	0.40	0.37	0.45	0.25	0.36	0.39	0.39	0.33	0.27	0.28	0.36	0.34	0.26	0.28	0.27	0.28	0.32	0.38	0.25	0.29	0.27	0.24	0.30	0.29	0.33
Total	100.0	100.0	100.0	100.0	100.0	100.0	100.0	100.0	100.0	100.0	100.0	100.0	100.0	100.0	100.0	100.0	100.0	100.0	100.0	100.0	100.0	100.0	100.0	100.0	100.0

*—other EU member states in 2024, i.e., Cyprus, Estonia, Finland, Ireland, Luxembourg, and Malta.

**Table A3:** Diversity of apple yields in European Union countries in 2000–2024.

Country	2000	2001	2002	2003	2004	2005	2006	2007	2008	2009	2010	2011	2012	2013	2014	2015	2016	2017	2018	2019	2020	2021	2022	2023	2024
Average yields in UE, in decitons per hectare	19.4	18.8	19.2	19.3	20.7	18.9	20.6	18.4	21.9	22.2	19.8	21.1	19.4	21.0	24.0	23.4	23.4	18.5	26.3	23.6	24.4	25.2	26.2	25.5	24.9
Average yields in the EU = 100																									
Austria	422.4	357.2	423.4	370.5	383.8	393.0	405.9	428.4	416.7	361.1	226.6	237.3	222.9	160.1	191.1	186.0	65.1	149.8	219.0	153.9	164.3	129.0	157.7	120.2	117.9
Belgium	316.7	203.4	211.6	196.5	207.3	193.4	202.2	228.8	188.5	181.5	225.3	139.7	159.7	153.4	186.7	177.3	156.9	75.5	146.9	177.1	125.4	185.3	173.9	162.2	143.5
Bulgaria	35.5	23.0	23.1	42.1	42.2	44.3	43.0	40.3	33.0	36.9	42.1	39.2	34.5	54.4	57.4	52.5	46.5	61.0	48.1	44.7	43.5	46.2	47.6	38.4	41.9
Croatia	79.0	24.7	43.0	38.5	58.1	54.0	47.8	57.1	40.8	51.0	68.4	72.1	32.2	107.7	67.7	75.7	32.5	63.1	72.6	58.5	59.7	54.0	48.9	66.0	71.2
Czechia	83.5	65.3	93.6	78.1	74.8	74.5	71.4	69.1	77.7	71.1	55.9	43.1	65.2	64.5	59.6	80.1	71.3	77.3	79.5	57.6	65.8	64.2	71.4	61.3	27.7
Denmark	76.9	59.6	62.4	102.2	106.7	115.7	100.6	117.0	88.0	84.6	76.1	80.6	71.5	107.4	106.8	109.9	117.2	86.3	87.5	76.9	118.6	86.7	55.0	35.3	50.6
France	207.6	198.5	210.6	183.0	183.8	207.4	186.1	223.8	184.3	188.9	216.7	210.4	173.6	213.6	153.3	169.7	155.4	181.9	131.0	147.6	130.4	119.6	126.0	137.9	149.5
Germany	129.7	135.1	140.5	135.8	146.5	146.0	141.8	183.3	150.0	151.4	132.8	134.8	158.1	120.8	147.6	132.7	140.9	94.9	134.2	123.7	123.2	117.4	123.3	111.3	105.8
Greece	115.2	95.2	107.4	70.2	99.3	99.2	105.6	103.4	88.1	89.0	103.1	97.5	102.8	91.3	107.0	116.4	123.4	146.9	105.1	119.6	86.4	110.8	103.6	97.5	116.2
Hungary	104.0	56.3	76.8	60.4	78.3	64.3	66.8	78.6	60.1	70.6	73.9	42.0	104.5	84.4	97.6	66.8	65.4	79.4	81.1	68.2	62.8	81.6	56.0	83.5	67.1
Italy	184.5	195.1	189.7	177.5	179.5	203.2	181.4	216.3	170.7	179.0	192.7	201.1	187.4	190.8	188.1	193.6	198.9	193.4	163.4	177.5	183.5	161.2	160.1	164.2	180.5
Latvia	22.5	23.5	32.0	22.6	4.0	23.3	17.5	22.5	25.6	13.9	16.0	10.5	15.5	25.4	15.1	13.8	17.1	12.1	16.5	12.8	16.2	10.2	12.5	9.1	18.8
Lithuania	16.4	25.7	15.0	26.2	8.5	24.6	32.6	16.6	29.0	20.7	14.2	18.4	25.9	20.9	18.1	23.8	22.9	38.1	30.3	13.4	20.2	14.2	19.8	16.7	29.5
Netherlands	185.6	185.2	165.4	180.4	206.4	195.3	185.7	226.0	183.7	200.6	194.8	239.8	181.9	188.9	187.2	189.3	184.7	176.1	155.1	180.3	145.2	162.9	152.5	140.9	148.4
Poland	45.4	77.8	67.2	78.8	69.6	64.8	69.2	32.2	75.0	68.1	55.8	64.4	76.0	75.9	75.4	75.2	86.9	74.7	91.6	83.9	95.3	99.7	107.0	101.6	91.9
Portugal	56.2	67.4	79.4	77.3	74.0	78.8	79.5	91.6	78.5	94.2	86.6	93.5	88.0	100.0	82.3	99.4	71.7	120.2	68.9	109.8	81.8	105.0	80.9	82.1	92.2
Romania	35.7	37.0	35.4	58.6	72.3	41.4	48.4	43.8	38.2	44.2	49.6	55.8	43.0	40.5	38.1	36.5	36.0	33.8	44.8	39.6	42.0	43.8	38.3	38.5	36.5
Slovakia	138.0	64.7	57.2	89.7	61.9	77.8	44.7	29.7	55.6	61.8	64.6	64.8	78.7	78.2	78.9	83.4	38.3	80.4	78.1	72.4	64.6	71.6	76.9	67.1	88.4
Slovenia	217.5	135.4	238.7	169.6	218.3	181.5	187.0	216.4	163.1	158.1	215.2	182.7	105.3	166.4	163.3	199.9	105.4	183.3	141.4	101.3	125.2	43.7	91.7	59.9	106.5
Spain	86.2	115.3	84.0	99.0	79.2	105.2	83.6	106.2	90.4	82.8	102.8	100.9	80.5	84.4	84.1	82.7	86.0	103.7	71.6	91.4	72.4	83.0	64.7	72.7	78.4
Sweden	79.6	74.6	70.5	83.4	64.8	65.0	73.0	81.5	72.3	63.0	79.3	74.9	80.2	93.1	79.3	81.6	72.9	85.3	82.6	61.9	83.4	87.6	82.9	86.9	87.4
Others *	27.4	18.1	20.9	12.3	17.4	22.9	24.7	26.3	22.2	21.0	33.1	31.3	25.5	29.2	28.3	30.0	36.4	43.4	48.6	55.4	52.5	46.0	56.7	51.9	60.2

*—other EU member states in 2024, i.e., Cyprus, Estonia, Finland, Ireland, Luxembourg, and Malta.

**Table A4:** The economic value of pollination of apple orchards in European Union countries (constant prices from 2024).

Country	2000	2001	2002	2003	2004	2005	2006	2007	2008	2009	2010	2011	2012	2013	2014	2015	2016	2017	2018	2019	2020	2021	2022	2023	2024
Total, million USD	6349.9	5325.6	5318.2	6539.1	7372.2	6446.7	7169.7	8713.6	9324.4	6870.4	6878.1	8054.2	7099.1	8786.0	7511.3	6651.1	6545.5	6224.2	8745.1	6290.8	7370.8	7503.7	6041.1	5793.9	6369.6
Share of total pollination value, in %																									
Austria	3.24	3.89	4.82	5.13	4.65	4.19	4.35	4.57	6.17	4.89	2.47	3.27	2.74	2.42	2.71	2.30	0.73	2.28	3.70	2.39	3.72	3.41	4.06	2.73	2.55
Belgium	3.53	3.51	2.98	4.12	3.92	3.54	4.50	3.65	4.13	3.07	3.48	2.77	2.67	2.70	3.29	2.35	1.78	0.94	2.58	1.87	1.79	2.82	1.79	1.94	1.59
Bulgaria	0.25	0.12	0.10	0.09	0.06	0.06	0.06	0.30	0.35	0.43	0.60	0.35	0.24	0.33	0.33	0.36	0.26	0.30	0.28	0.29	0.22	0.26	0.30	0.21	0.20
Croatia	0.39	0.19	0.28	0.34	0.54	0.55	0.44	0.43	0.42	0.51	0.59	0.59	0.29	0.47	0.40	0.48	0.29	0.42	0.34	0.39	0.51	0.46	0.34	0.45	0.47
Czechia	1.73	1.58	1.28	1.11	1.10	1.15	1.23	0.70	0.58	0.49	0.50	0.43	0.58	0.67	0.76	0.81	0.67	0.86	0.74	0.66	0.76	0.78	1.34	1.02	0.40
Denmark	0.27	0.27	0.28	0.55	0.60	0.48	0.47	0.44	0.24	0.26	0.23	0.25	0.20	0.25	0.42	0.32	0.30	0.17	0.22	0.21	0.43	0.33	0.26	0.17	0.23
France	13.53	16.85	16.55	14.95	16.48	16.81	19.13	18.50	16.73	18.34	20.70	17.12	18.89	19.21	20.41	22.43	22.22	21.41	17.78	21.14	19.18	20.16	22.49	25.58	23.29
Germany	25.71	18.40	17.16	11.35	12.14	10.05	10.81	11.35	12.27	12.01	9.57	10.98	11.63	9.74	11.55	8.60	10.20	7.86	13.46	9.71	11.74	11.58	10.55	9.30	9.68
Greece	2.83	3.13	4.04	3.21	3.96	3.57	3.79	3.13	3.50	4.55	4.10	3.67	3.67	2.95	3.36	3.27	3.16	3.17	3.00	3.56	3.58	3.56	3.70	3.11	3.31
Hungary	2.85	1.96	1.43	2.04	1.72	3.00	2.86	5.29	1.39	1.16	2.42	1.36	2.51	1.80	1.37	2.00	1.30	2.96	1.28	2.08	1.59	1.23	1.24	1.22	1.43
Italy	13.66	18.16	21.53	18.66	20.05	18.83	17.58	18.51	20.89	24.18	21.95	19.41	21.37	23.03	22.56	21.45	26.27	21.58	25.26	23.83	21.48	20.51	21.83	20.25	22.24
Latvia	0.12	0.11	0.18	0.14	0.07	0.16	0.24	0.22	0.22	0.09	0.12	0.05	0.10	0.07	0.08	0.06	0.09	0.09	0.15	0.16	0.20	0.11	0.18	0.12	0.17
Lithuania	0.22	0.16	0.11	1.02	0.33	0.75	0.41	0.53	0.83	0.45	0.41	0.62	0.80	0.48	0.66	0.73	0.60	0.89	0.70	0.30	0.55	0.36	0.63	0.40	0.67
Netherlands	2.82	3.59	1.82	2.32	2.77	3.03	3.45	3.69	4.24	3.95	3.78	5.15	3.88	4.40	4.27	3.37	3.22	3.46	3.70	3.43	2.81	3.64	2.42	2.41	2.72
Poland	2.98	4.62	4.64	8.48	6.23	9.84	9.58	8.07	8.96	6.60	9.44	13.76	13.54	14.30	10.12	14.50	12.04	15.35	10.66	11.36	14.36	11.03	11.67	13.05	15.59
Portugal	2.82	3.50	3.71	3.39	3.67	4.05	3.18	3.07	3.10	3.84	3.18	3.55	3.10	3.67	3.46	3.76	3.16	5.00	2.91	5.20	3.66	5.11	4.27	4.17	3.84
Romania	15.21	10.00	9.02	11.05	13.02	11.44	10.57	8.90	8.33	8.42	8.38	9.47	7.20	6.53	7.64	6.55	6.88	5.55	6.92	6.12	7.08	7.22	6.68	6.50	5.04
Slovakia	0.63	0.48	0.50	0.57	0.35	0.45	0.27	0.16	0.39	0.42	0.43	0.34	0.48	0.36	0.43	0.37	0.18	0.36	0.31	0.32	0.26	0.32	0.39	0.32	0.41
Slovenia	1.21	0.89	1.51	1.19	1.24	1.12	1.15	1.26	1.41	1.20	1.36	1.16	0.64	1.04	0.92	1.14	0.64	1.14	0.55	0.57	0.67	0.28	0.60	0.39	0.57
Spain	5.77	8.35	7.81	10.03	6.91	6.66	5.61	6.98	5.55	4.87	5.87	5.38	5.03	5.19	4.89	4.77	5.65	5.79	5.03	5.98	4.80	6.27	4.82	6.30	5.19
Sweden	0.22	0.25	0.24	0.27	0.21	0.26	0.33	0.27	0.30	0.30	0.42	0.32	0.44	0.38	0.39	0.36	0.34	0.42	0.43	0.43	0.61	0.56	0.45	0.37	0.42
Total	100.00	100.00	100.00	100.00	100.00	100.00	100.00	100.00	100.00	100.00	100.00	100.00	100.00	100.00	100.00	100.00	100.00	100.00	100.00	100.00	100.00	100.00	100.00	100.00	100.00

**Table A5:** Economic value of pollination of 1 hectare of apple orchard in European Union countries (constant 2024 prices), in thousand USD per ha.

Country	2000	2001	2002	2003	2004	2005	2006	2007	2008	2009	2010	2011	2012	2013	2014	2015	2016	2017	2018	2019	2020	2021	2022	2023	2024
Average	8.95	8.00	8.70	11.40	12.63	11.11	13.35	16.05	17.55	13.21	13.32	15.44	13.37	16.40	14.41	12.73	12.72	12.08	17.33	12.88	15.14	15.31	12.70	12.35	13.83
Austria	34.26	33.96	43.41	56.88	56.14	44.30	51.14	65.64	95.37	55.58	28.04	43.58	32.10	30.45	30.06	23.16	7.20	21.27	48.02	22.84	42.63	40.32	38.93	25.33	27.18
Belgium	25.22	21.24	18.42	32.08	34.78	25.64	37.48	37.43	47.57	27.44	30.94	28.83	26.72	33.36	34.85	22.79	17.96	9.49	37.64	20.32	24.10	39.55	20.63	22.90	22.54
Bulgaria	1.24	0.67	0.92	1.18	0.95	1.29	1.49	7.30	10.10	6.84	7.92	5.83	3.66	6.08	6.34	5.02	4.20	4.69	6.15	4.35	4.56	5.16	4.91	3.39	3.57
Croatia	5.92	2.10	2.78	3.60	7.64	6.35	5.33	6.23	6.05	5.34	6.15	7.27	3.46	7.76	5.00	5.53	3.23	5.37	6.30	4.97	8.61	7.89	5.62	7.18	7.74
Czechia	5.24	4.69	7.36	7.76	8.51	7.77	9.53	6.72	5.93	3.72	3.78	3.68	4.37	6.57	6.37	6.51	5.87	7.27	8.93	5.67	7.76	8.25	11.57	9.12	4.73
Denmark	10.16	8.01	9.58	22.12	27.64	19.10	21.94	25.69	15.11	12.02	9.95	13.33	10.64	16.14	23.08	15.09	14.66	8.32	13.73	9.72	22.74	17.92	10.46	6.74	9.78
France	16.21	17.80	17.79	20.10	25.65	23.20	31.25	37.26	37.09	30.58	34.80	34.71	34.63	44.89	30.56	30.04	29.00	26.49	30.77	26.41	25.84	27.91	25.15	27.55	28.11
Germany	13.06	14.00	16.70	23.82	27.66	20.03	23.84	31.18	35.99	25.93	20.69	27.99	26.10	27.04	27.57	18.22	21.31	14.43	34.63	17.98	25.47	25.56	19.25	16.27	18.63
Greece	12.75	12.05	15.71	13.72	21.83	17.24	20.42	20.64	24.29	23.03	20.99	22.17	19.59	19.59	25.74	22.13	21.34	21.25	25.37	22.82	18.35	25.98	20.99	18.48	23.16
Hungary	5.24	1.83	2.12	3.07	2.93	4.60	5.24	11.38	3.01	2.17	4.89	3.30	5.57	4.75	3.09	4.06	2.62	5.73	3.51	4.22	4.50	3.68	3.14	3.10	4.04
Italy	13.87	15.44	18.91	21.43	25.68	21.24	22.06	28.80	33.01	28.43	26.07	27.50	27.74	36.61	30.95	26.07	32.58	25.06	38.46	27.26	28.83	28.25	24.54	21.70	26.53
Latvia	0.93	0.73	1.17	1.09	0.59	1.24	1.80	2.57	3.94	1.41	2.56	1.28	2.25	2.31	2.19	1.74	2.37	1.73	4.14	2.84	4.19	2.62	3.65	2.02	3.53
Lithuania	0.45	0.27	0.22	3.48	1.27	2.34	1.98	3.44	6.67	2.66	2.30	3.91	3.97	2.94	3.57	3.67	3.22	4.48	6.03	1.84	3.89	2.63	3.88	2.57	5.09
Netherlands	13.96	16.30	8.67	14.73	20.00	20.05	25.85	34.19	42.55	29.71	29.91	50.15	34.62	48.94	40.90	29.47	28.77	30.96	49.04	33.64	33.45	45.73	24.74	25.39	33.05
Poland	1.15	1.48	1.47	3.48	2.62	3.74	4.24	4.01	4.86	2.61	3.81	6.04	4.94	6.50	4.31	5.35	4.45	5.42	5.61	4.59	6.94	5.11	4.64	5.04	6.71
Portugal	8.48	9.13	9.99	11.61	15.08	15.50	14.52	18.38	21.01	21.01	17.59	22.81	17.08	23.59	18.76	17.86	14.37	21.04	17.44	22.86	18.87	27.55	18.78	17.33	17.93
Romania	13.60	7.29	6.62	10.09	13.08	9.03	12.78	13.14	14.19	10.99	10.22	14.47	9.23	9.52	10.23	7.79	8.11	6.22	11.21	7.30	9.98	10.06	7.46	6.93	5.99
Slovakia	13.08	6.14	5.72	10.60	7.90	9.12	5.76	4.34	10.68	10.42	11.12	11.87	11.75	11.36	12.55	10.45	5.12	10.31	12.78	9.67	10.61	14.79	15.16	12.09	16.99
Slovenia	24.97	15.32	25.96	25.14	29.40	23.29	26.64	38.21	45.70	30.19	33.78	34.13	16.90	34.46	27.06	30.70	17.28	30.02	20.77	15.75	22.76	9.93	17.82	11.26	18.87
Spain	7.52	10.51	9.61	14.25	12.07	11.01	10.63	16.47	15.51	10.22	12.70	13.75	11.60	14.81	11.94	10.34	11.99	11.79	14.70	12.69	12.00	15.99	9.96	12.85	11.82
Sweden	9.88	9.29	9.60	13.22	11.20	11.68	14.97	16.60	20.16	13.90	19.20	19.60	20.61	24.09	22.45	18.25	14.51	18.76	26.49	17.77	31.18	28.68	18.62	14.73	18.67

## References

[1] Costanza R., De Groot R., Sutton P., Van Der Ploeg S., Anderson S.J., Kubiszewski I., Farber S., Turner R.K. (2014). Changes in the Global Value of Ecosystem Services. Glob. Environ. Change.

[2] Costanza R., De Groot R., Braat L., Kubiszewski I., Fioramonti L., Sutton P., Farber S., Grasso M. (2017). Twenty Years of Ecosystem Services: How Far Have We Come and How Far Do We Still Need to Go?. Ecosyst. Serv..

[3] Abd Elbasit M.A., Knight J., Liu G., Abu-Zreig M.M., Hasaan R. (2021). Valuation of Ecosystem Services in South Africa, 2001–2019. Sustainability.

[4] Faber A., Jarosz Z. (2023). Charakterystyka Zrównoważenia Rozwoju Biogospodarki w Polsce-Wymiar Ekologiczny. Zesz. Nauk. Szk. Gł. Gospod. Wiej. Warszawie. Probl. Rol. Świat..

[5] Guth M., Smędzik-Ambroży K. (2017). Zasoby a Zrównoważony Rozwój Rolnictwa w Polsce Po Integracji z UE. Zesz. Nauk. Szk. Gł. Gospod. Wiej. Warszawie. Probl. Rol. Świat..

[6] Kozar Ł. (2017). Rozwój Zielonej Gospodarki w Sektorze Rolnictwa w Krajach Unii Europejskiej i w Polsce w Kontekście Koncepcji Zrównoważonego Rozwoju. PRS.

[7] Power A.G. (2010). Ecosystem Services and Agriculture: Tradeoffs and Synergies. Phil. Trans. R. Soc. B.

[8] Swinton S.M., Lupi F., Robertson G.P., Hamilton S.K. (2007). Ecosystem Services and Agriculture: Cultivating Agricultural Ecosystems for Diverse Benefits. Ecol. Econ..

[9] Asah S.T., Guerry A.D., Blahna D.J., Lawler J.J. (2014). Perception, Acquisition and Use of Ecosystem Services: Human Behavior, and Ecosystem Management and Policy Implications. Ecosyst. Serv..

[10] Coutts C., Hahn M. (2015). Green Infrastructure, Ecosystem Services, and Human Health. Int. J. Environ. Res. Public Health.

[11] Kosanic A., Petzold J. (2020). A Systematic Review of Cultural Ecosystem Services and Human Wellbeing. Ecosyst. Serv..

[12] Pejchar L., Mooney H.A. (2009). Invasive Species, Ecosystem Services and Human Well-Being. Trends Ecol. Evol..

[13] Popławski Ł., Kaczmarczyk B. (2016). Problemy Zrównoważonego Rozwoju-Wycena Przestrzeni Publicznej. Res. Pap. Wroc. Univ. Econ./Pr. Nauk. Uniw. Ekon. Wroclawiu.

[14] Costanza R., d’Arge R., De Groot R., Farber S., Grasso M., Hannon B., Limburg K., Naeem S., O’neill R.V., Paruelo J. (1997). The Value of the World’s Ecosystem Services and Natural Capital. Nature.

[15] Hanson C., Ranganathan J., Iceland C., Finisdore J. (2012). The Corporate Ecosystem Services Review: Guidelines for Identifying Business Risk and Opportunities Arising from Ecosystem Change. Version 2.0.

[16] Lele S., Springate-Baginski O., Lakerveld R., Deb D., Dash P. (2013). Ecosystem Services: Origins, Contributions, Pitfalls, and Alternatives. Conserv. Soc..

[17] Jaroszewska J., Prandecki K. (2016). Znaczenie “Zazielenienia” w Zapewnieniu Różnorodności Biologicznej. Zesz. Nauk. Szk. Gł. Gospod. Wiej. Warszawie. Probl. Rol. Świat..

[18] Hermoso V., Carvalho S.B., Giakoumi S., Goldsborough D., Katsanevakis S., Leontiou S., Markantonatou V., Rumes B., Vogiatzakis I.N., Yates K.L. (2022). The EU Biodiversity Strategy for 2030: Opportunities and Challenges on the Path towards Biodiversity Recovery. Environ. Sci. Policy.

[19] Landis D.A. (2017). Designing Agricultural Landscapes for Biodiversity-Based Ecosystem Services. Basic Appl. Ecol..

[20] Kubiszewski I., Costanza R., Anderson S., Sutton P. (2017). The Future Value of Ecosystem Services: Global Scenarios and National Implications. Ecosyst. Serv..

[21] Tohidyan Far S., Rezaei-Moghaddam K. (2019). Multifunctional Agriculture: An Approach for Entrepreneurship Development of Agricultural Sector. J. Glob. Entrep. Res..

[22] Eftekhari H., Shadparvar V. (2018). Multifunctional Agriculture: Agriculture Renewal as a Modern Paradigm for Agriculture and Rural Development. Int. J. Agric. Manag. Dev..

[23] Czyżewski A., Kułyk P. (2011). Dobra Publiczne w Koncepcji Wielofunkcyjnego Rozwoju Rolnictwa, Ujęcie Teoretyczne i Praktyczne. Zesz. Nauk. Szk. Gł. Gospod. Wiej. Warszawie. Probl. Rol. Świat..

[24] Dokurno Z., Fiedor B., Scheuer B. (2016). Makroekonomiczna i metodologiczna perspektywa dyskursu wokół pojęcia zielonej gospodarki. Gospod. Nar. Pol. J. Econ..

[25] Saunders M.E. (2018). Insect Pollinators Collect Pollen from Wind-pollinated Plants: Implications for Pollination Ecology and Sustainable Agriculture. Insect. Conserv. Divers..

[26] Nogué S., Long P.R., Eycott A.E., De Nascimento L., Fernández-Palacios J.M., Petrokofsky G., Vandvik V., Willis K.J. (2016). Pollination Service Delivery for European Crops: Challenges and Opportunities. Ecol. Econ..

[27] Isaacs R., Williams N., Ellis J., Pitts-Singer T.L., Bommarco R., Vaughan M. (2017). Integrated Crop Pollination: Combining Strategies to Ensure Stable and Sustainable Yields of Pollination-Dependent Crops. Basic Appl. Ecol..

[28] Kevan P.G., Clark E.A., Thomas V.G. (1990). Insect Pollinators and Sustainable Agriculture. Am. J. Altern. Agric..

[29] Süle G., Báldi A., Kleijn D., Steffan-Dewenter I., Albrecht M., Batáry P., Biegerl C., Bogusch P., Braunisch V., Buse J. (2026). Synthesis of Pollinator-Friendly Management in European Protected Areas. Conserv. Biol..

[30] Ollerton J., Winfree R., Tarrant S. (2011). How Many Flowering Plants Are Pollinated by Animals?. Oikos.

[31] Peña-Kairath C., Delclòs X., Álvarez-Parra S., Peñalver E., Engel M.S., Ollerton J., Peris D. (2023). Insect Pollination in Deep Time. Trends Ecol. Evol..

[32] Ollerton J. (2017). Pollinator Diversity: Distribution, Ecological Function, and Conservation. Annu. Rev. Ecol. Evol. Syst..

[33] Potts S.G., Biesmeijer J.C., Kremen C., Neumann P., Schweiger O., Kunin W.E. (2010). Global Pollinator Declines: Trends, Impacts and Drivers. Trends Ecol. Evol..

[34] Brittain C., Benke S., Pecze R., Potts S.G., Peris-Felipo F.J., Vasileiadis V.P. (2022). Flower Margins: Attractiveness over Time for Different Pollinator Groups. Land.

[35] Schulp C.J.E., Lautenbach S., Verburg P.H. (2014). Quantifying and Mapping Ecosystem Services: Demand and Supply of Pollination in the European Union. Ecol. Indic..

[36] Winfree R., Gross B.J., Kremen C. (2011). Valuing Pollination Services to Agriculture. Ecol. Econ..

[37] Madras-Majewska B., Majewski J. Importance of Bees in Pollination of Crops in the European Union Countries. Proceedings of the Economic Science for Rural Development Conference Proceedings.

[38] Bishop G.A., Kleijn D., Albrecht M., Bartomeus I., Isaacs R., Kremen C., Magrach A., Ponisio L.C., Potts S.G., Scheper J. (2025). Critical Habitat Thresholds for Effective Pollinator Conservation in Agricultural Landscapes. Science.

[39] Gallai N., Salles J.-M., Settele J., Vaissière B.E. (2009). Economic Valuation of the Vulnerability of World Agriculture Confronted with Pollinator Decline. Ecol. Econ..

[40] Garratt M.P.D., Breeze T.D., Boreux V., Fountain M.T., McKerchar M., Webber S.M., Coston D.J., Jenner N., Dean R., Westbury D.B. (2016). Apple Pollination: Demand Depends on Variety and Supply Depends on Pollinator Identity. PLoS ONE.

[41] Klein A.-M., Vaissière B.E., Cane J.H., Steffan-Dewenter I., Cunningham S.A., Kremen C., Tscharntke T. (2007). Importance of Pollinators in Changing Landscapes for World Crops. Proc. R. Soc. B Biol. Sci..

[42] Lautenbach S., Seppelt R., Liebscher J., Dormann C.F. (2012). Spatial and Temporal Trends of Global Pollination Benefit. PLoS ONE.

[43] Liu Q., Xu P., Yan K., Guo Y. (2019). Pollination Services from Insects in Homegardens in the Chengdu Plain Will Be Confronted with Crises. Sustainability.

[44] Khalifa S.A., Elshafiey E.H., Shetaia A.A., El-Wahed A.A.A., Algethami A.F., Musharraf S.G., AlAjmi M.F., Zhao C., Masry S.H., Abdel-Daim M.M. (2021). Overview of Bee Pollination and Its Economic Value for Crop Production. Insects.

[45] Chaplin-Kramer R., Dombeck E., Gerber J., Knuth K.A., Mueller N.D., Mueller M., Ziv G., Klein A.-M. (2014). Global Malnutrition Overlaps with Pollinator-Dependent Micronutrient Production. Proc. R. Soc. B Biol. Sci..

[46] Aizen M.A., Garibaldi L.A., Cunningham S.A., Klein A.M. (2008). Long-Term Global Trends in Crop Yield and Production Reveal No Current Pollination Shortage but Increasing Pollinator Dependency. Curr. Biol..

[47] Morse R.A., Calderone N.W. (2000). The Value of Honey Bees as Pollinators of US Crops in 2000. Bee Cult..

[48] Majewski J. (2018). Ecosystem Services Value: Case of Pollination. Proceedings of the 2018 International Conference Economic Science for Rural Development, Jelgava, Latvia, 9–11 May 2018.

[49] Veldtman R., De Lange W.J. (2025). Valuing Insect Pollination Services to Safeguard Crop Pollination in South Africa. Insects.

[50] Kearns C.A., Inouye D.W., Waser N.M. (1998). Endangered Mutualisms: The Conservation of Plant-Pollinator Interactions. Annu. Rev. Ecol. Syst..

[51] Kremen C., Williams N.M., Aizen M.A., Gemmill-Herren B., LeBuhn G., Minckley R., Packer L., Potts S.G., Roulston T., Steffan-Dewenter I. (2007). Pollination and Other Ecosystem Services Produced by Mobile Organisms: A Conceptual Framework for the Effects of Land-use Change. Ecol. Lett..

[52] Porto R.G., De Almeida R.F., Cruz-Neto O., Tabarelli M., Viana B.F., Peres C.A., Lopes A.V. (2020). Pollination Ecosystem Services: A Comprehensive Review of Economic Values, Research Funding and Policy Actions. Food Secur..

[53] Leonhardt S.D., Gallai N., Garibaldi L.A., Kuhlmann M., Klein A.-M. (2013). Economic Gain, Stability of Pollination and Bee Diversity Decrease from Southern to Northern Europe. Basic Appl. Ecol..

[54] Reilly J.R., Artz D.R., Biddinger D., Bobiwash K., Boyle N.K., Brittain C., Brokaw J., Campbell J.W., Daniels J., Elle E. (2020). Crop Production in the USA Is Frequently Limited by a Lack of Pollinators. Proc. R. Soc. B Biol. Sci..

[55] Jordan A., Patch H.M., Grozinger C.M., Khanna V. (2021). Economic Dependence and Vulnerability of United States Agricultural Sector on Insect-Mediated Pollination Service. Environ. Sci. Technol..

[56] Majewski J., Popa D., Sobolewska A. (2021). Economic Value of Pollination of Orchards, Case of Moldova and Poland. Sci. Pap. Ser. Manag. Econ. Eng. Agric. Rural. Dev..

[57] Zych M., Jakubiec A. (2006). How Much Is Bee Worth? Economic Aspects of Pollination of Selected Crops in Poland. Acta Agrobot..

[58] Chen Y., Zhao Z., Zhang S. (2026). Economic Value, Relative Vulnerability and Honeybee Colony Supply–Demand Imbalance in Chinese Agriculture, 2010–2024. Insects.

[59] Hanley N., Breeze T.D., Ellis C., Goulson D. (2015). Measuring the Economic Value of Pollination Services: Principles, Evidence and Knowledge Gaps. Ecosyst. Serv..

[60] Breeze T.D., Gallai N., Garibaldi L.A., Li X.S. (2016). Economic Measures of Pollination Services: Shortcomings and Future Directions. Trends Ecol. Evol..

[61] Majewski J. (2018). Pollination Value as an Ecosystem Service. Ekon. I Sr..

[62] Mburu J., Hein L.G., Gemmill B., Collette L. (2006). Economic Valuation of Pollination Services: Review of Methods.

[63] Hoshide A.K., Drummond F.A., Stevens T.H., Venturini E.M., Hanes S.P., Sylvia M.M., Loftin C.S., Yarborough D.E., Averill A.L. (2018). What Is the Value of Wild Bee Pollination for Wild Blueberries and Cranberries, and Who Values It?. Environments.

[64] Majewski J. (2014). Economic Value of Pollination of Major Crops in Poland. Econ. Sci. Rural. Dev..

[65] Taste the Season: Eat More Fruit and Vegetables-Agriculture and Rural Development. https://agriculture.ec.europa.eu/farming/crops/fruit-and-vegetables/taste-season-eat-more-fruit-and-vegetables_en.

[66] Muder A., Garming H., Dreisiebner-Lanz S., Kerngast K., Rosner F., Kličková K., Kurthy G., Cimer K., Bertazzoli A., Altamura V. (2022). Apple Production and Apple Value Chains in Europe. Eur. J. Hortic. Sci..

[67] Bugała A. (2014). Światowy Rynek Jabłek i Zagęszczonego Soku Jabłkowego. Zesz. Nauk. Szk. Gł. Gospod. Wiej. Warszawie Probl. Rol. Swiat..

[68] Losey J.E., Vaughan M. (2006). The Economic Value of Ecological Services Provided by Insects. Bioscience.

[69] Melathopoulos A.P., Cutler G.C., Tyedmers P. (2015). Where Is the Value in Valuing Pollination Ecosystem Services to Agriculture?. Ecol. Econ..

[70] Giannini T.C., Cordeiro G.D., Freitas B.M., Saraiva A.M., Imperatriz-Fonseca V.L. (2015). The Dependence of Crops for Pollinators and the Economic Value of Pollination in Brazil. J. Econ. Entomol..

[71] FAOSTAT. https://www.fao.org/faostat/en/#home.

[72] Hein L. (2009). The Economic Value of the Pollination Service, a Review across Scales. Open Ecol. J..

[73] Paudel Y.P., Mackereth R., Hanley R., Qin W. (2015). Honey Bees (*Apis mellifera* L.) and Pollination Issues: Current Status, Impacts and Potential Drivers of Decline. J. Agric. Sci..

[74] Jacquemin F., Violle C., Rasmont P., Dufrêne M. (2017). Mapping the Dependency of Crops on Pollinators in Belgium. One Ecosyst..

[75] Bugin G., Lenzi L., Ranzani G., Barisan L., Porrini C., Zanella A., Bolzonella C. (2022). Agriculture and Pollinating Insects, No Longer a Choice but a Need: EU Agriculture’s Dependence on Pollinators in the 2007–2019 Period. Sustainability.

[76] Ramírez F., Davenport T.L. (2013). Apple Pollination: A Review. Sci. Hortic..

[77] Pardo A., Borges P.A. (2020). Worldwide Importance of Insect Pollination in Apple Orchards: A Review. Agric. Ecosyst. Environ..

[78] Riva C., Gachoki S., Adjlane N., Bevk D., Haider Y., Hatjina F., Kolega Š., Kovačić M., Manthos I., Puškadija Z. (2026). Climate Impacts Apple Pollination, Yield and Economic Outcomes of Farmers. Sci. Total Environ..

[79] Burns K.L.W., Stanley D.A. (2022). The Importance and Value of Insect Pollination to Apples: A Regional Case Study of Key Cultivars. Agric. Ecosyst. Environ..

[80] Olhnuud A., Liu Y., Makowski D., Tscharntke T., Westphal C., Wu P., Wang M., Van Der Werf W. (2022). Pollination Deficits and Contributions of Pollinators in Apple Production: A Global Meta-analysis. J. Appl. Ecol..

[81] Eeraerts M., Osterman J., Batáry P., Klein A., Albrecht M., Andersson G.K.S., Báldi A., Bernauer O.M., Blechschmidt L., Blitzer E.J. (2025). Global Synthesis of Apple Pollination Research Highlights General Pollen Limitation and Positive Contributions of Wild Bees Compared to Honeybees. J. Appl. Ecol..

[82] Fraser A.M., Allington G.R.H., Luthra A., Virkar P., Rana S., Suyal R., Rawat A., Ratna A., Cunningham K., Raikwal N. (2024). Pollination Deficit in Apples Increases with Elevation. Agric. Ecosyst. Environ..

[83] Elsysy M., Ebrahimi A., Einhorn T. (2025). Wind Pollination of Apple Flowers under Insect Exclusion Nets Questions the Insect-Dependent Pollination Model of Modern Apple Plantations. Plants.

[84] Mupepele A., Von Königslöw V., Bleile A., Fornoff F., Fründ J., Klein A. (2025). Plant–Pollinator Interactions in Apple Orchards from a Production and Conservation Perspective. Conserv. Sci. Pract..

[85] Calderone N.W. (2012). Insect Pollinated Crops, Insect Pollinators and US Agriculture: Trend Analysis of Aggregate Data for the Period 1992–2009. PLoS ONE.

[86] Földesi R., Kovács-Hostyánszki A., Kőrösi Á., Somay L., Elek Z., Markó V., Sárospataki M., Bakos R., Varga Á., Nyisztor K. (2016). Relationships between Wild Bees, Hoverflies and Pollination Success in Apple Orchards with Different Landscape Contexts. Agri. For. Entomol..

[87] Roquer-Beni L., Arnan X., Rodrigo A., Bosch J. (2022). What Makes a Good Pollinator? Relationship between Pollinator Traits and Pollination Effectiveness in Apple Flowers. Entomologia.

[88] Vassvik L., Nielsen A., Garratt M., Hatteland B.A., Chipperfield J., Johansen J., Høydal S.M.M., Aschehoug E. (2026). Pollination Success in Apples Is Dependent upon Wild Bees and Orchard Design. J. Pollinat. Ecol..

[89] Sapir G., Baras Z., Azmon G., Goldway M., Shafir S., Allouche A., Stern E., Stern R.A. (2017). Synergistic Effects between Bumblebees and Honey Bees in Apple Orchards Increase Cross Pollination, Seed Number and Fruit Size. Sci. Hortic..

[90] Barda M., Karamaouna F., Kati V., Perdikis D. (2023). Do Patches of Flowering Plants Enhance Insect Pollinators in Apple Orchards?. Insects.

[91] Garratt M.P.D., O’Connor R.S., Carvell C., Fountain M.T., Breeze T.D., Pywell R., Redhead J.W., Kinneen L., Mitschunas N., Truslove L. (2023). Addressing Pollination Deficits in Orchard Crops through Habitat Management for Wild Pollinators. Ecol. Appl..

[92] Bhatia S., Kumari N., Sharma S., Kumar L., Thakur Y., Garg A., Chauhan K., Kumari N., Sharma S., Kumar N., Banyal H.S. (2024). Guardians of the Orchard: The Vital Role of Pollinators in Apple Production. Insect Pollinators Diversity, Distribution, Ecological Function and Conservation Status in Himalayan Region.

[93] Radzevičiūtė R., Theodorou P., Schlegel M., Paxton R.J. (2021). A Two-Part Modelling Approach Reveals a Positive Effect of Pollinator Biodiversity in Boosting the Pollination of Apple Flowers. Agric. Ecosyst. Environ..

[94] Russo L., Park M.G., Blitzer E.J., Danforth B.N. (2017). Flower Handling Behavior and Abundance Determine the Relative Contribution of Pollinators to Seed Set in Apple Orchards. Agric. Ecosyst. Environ..

[95] Fountain M.T., Tsiolis K., Silva C.X., Deakin G., Garratt M.P.D., O’Connor R., Carvell C., Pywell R.F., Edwards M., Potts S.G. (2023). Location and Creation of Nest Sites for Ground-Nesting Bees in Apple Orchards. Insects.

[96] Li J., Olhnuud A., Tscharntke T., Wang M., Wu P., Xu H., Liu Y. (2025). Honeybees Interfere with Wild Bees in Apple Pollination in China. J. Appl. Ecol..

[97] Viana B.F., da Encarnação Coutinho J.G., Garibaldi L.A., Castagnino G.L.B., Gramacho K.P., Silva F.O. (2014). Stingless Bees Further Improve Apple Pollination and Production. J. Pollinat. Ecol..

[98] Wilcox S.W., Just D.R., Grab H., Lawell C.-Y., Yeh D.A., Wootan S. (2025). Apple Pollination Costs and Strategies: Trends and Outlook. Choices.

[99] Vassvik L., Nielsen A., Garratt M.P.D., Hatteland B.A., Chipperfield J.D., Aschehoug E.T. (2026). Alternate Floral Resources within Apple Orchards Increase Apple Flower Visitation. J. Insect. Conserv..

[100] Fliszkiewicz M., Giejdasz K. (2023). Effect of Pollination by the *Osmia bicornis* (Syn. *O. rufa*) Bee on Fruit Set, Seed Set and Yield in Three Apple Cultivars. J. Apic. Sci..

[101] Pizło W. (2014). Perspektywy rozwoju gospodarstw jabłoniowych w Polsce na tle wybranych krajów UE. Zesz. Nauk. SGGW Warszawie Probl. Rol. Świat..

[102] Jąder K. (2018). Produkcja, konsumpcja i eksport jabłek w Polsce w latach 2005–2016. Zesz. Nauk. SGGW Warszawie Probl. Rol. Świat..

[103] Filyova P., Ivanov P. (2025). Status and Trends in the Development of Apple Production. J. Mt. Agric. Balk..

[104] Muder A., Staniszewski J. (2025). Efficiency and Technology Gap in European Apple Production—A Metafrontier Model for Germany, Italy, and Poland. Agribusiness.

[105] Minga A., Marku D. (2023). Determinants of Technical Efficiency and Production Costs in Small-Scale Apple Farms in Albania. Univers. J. Agric. Res..

[106] Szymańska E.J., Rysz M. (2022). Determinants of Changes in Fruit Production in Farms in Areas with a Fragmented Agrarian Structure. Agriculture.

[107] Badiu D., Arion F., Muresan I., Lile R., Mitre V. (2015). Evaluation of Economic Efficiency of Apple Orchard Investments. Sustainability.

[108] Larue B., Ker A.P. (2024). Climate Change, Production and Trade in Apples. Can. J. Agri. Econ..

[109] Rysz M., Szymańska E. (2023). Wdrażanie Innowacji w Gospodarstwach Sadowniczych o Różnej Skali Produkcji Owoców. Ekon. Organ. Logistyki.

[110] Manzoor S.H., Zhang Z., Yu S., Zhang Z., Zhu D., Zuo C., Han B., Liu P., Wang Z. (2025). Precision Pollination in Apple Orchards: A Pollination Model for Calculating Importance of Apple Flowering Stages for Yield Maximization. Apple Production Technologies: From Laboratory to Practical Applications.

[111] Schmitz C., Zimmermann L., Schiffers K., Balmer M., Luedeling E. (2025). ProbApple—A Probabilistic Model to Forecast Apple Yield and Quality. Agric. Syst..

[112] Geslin B., Aizen M.A., Garcia N., Pereira A.-J., Vaissière B.E., Garibaldi L.A. (2017). The Impact of Honey Bee Colony Quality on Crop Yield and Farmers’ Profit in Apples and Pears. Agric. Ecosyst. Environ..

[113] Cebotari V., Buzu I. (2014). Bee Colonies Exploitation at Apple Blossom Pollination in the Orchards. Sci. Pap. Ser. D. Anim. Sci..

[114] Roquer-Beni L., Alins G., Arnan X., Boreux V., García D., Hambäck P.A., Happe A., Klein A., Miñarro M., Mody K. (2021). Management-dependent Effects of Pollinator Functional Diversity on Apple Pollination Services: A Response–Effect Trait Approach. J. Appl. Ecol..

[115] McKerchar M., Potts S.G., Fountain M.T., Garratt M.P.D., Westbury D.B. (2020). The Potential for Wildflower Interventions to Enhance Natural Enemies and Pollinators in Commercial Apple Orchards Is Limited by Other Management Practices. Agric. Ecosyst. Environ..

[116] Blitzer E.J., Gibbs J., Park M.G., Danforth B.N. (2016). Pollination Services for Apple Are Dependent on Diverse Wild Bee Communities. Agric. Ecosyst. Environ..

[117] Demestihas C., Plénet D., Génard M., Raynal C., Lescourret F. (2017). Ecosystem Services in Orchards. A Review. Agron. Sustain. Dev..

[118] Vizzotto G., Driussi E., Pontoni M., Testolin R. (2018). Effect of Flower Pollination on Fruit Set and Cropping in Apple. Am. J. Agric. For..

[119] Broussard M.A., Coates M., Martinsen P. (2023). Artificial Pollination Technologies: A Review. Agronomy.

[120] Rahimi E., Jung C. (2024). Global Trends in Climate Suitability of Bees: Ups and Downs in a Warming World. Insects.

[121] Blili Y., Abou Nader E., Pavez I., Prosperi P., Harbouze R., Kyrgiakos L.S., Kleisiari C., Vasileiou M., Angelopoulos V., Vlontzos G. (2025). Assessing the Economic Impact of Insect Pollination on the Agricultural Sector: A Department-Level Case Study in France. Environ. Sustain. Indic..

[122] Garratt M.P.D., Truslove L., Coston D., Evans R., Moss E., Dodson C., Jenner N., Biesmeijer J., Potts S. (2014). Pollination Deficits in UK Apple Orchards. J. Pollinat. Ecol..

[123] Garratt M.P., Breeze T.D., Jenner N., Polce C., Biesmeijer J.C., Potts S.G. (2014). Avoiding a Bad Apple: Insect Pollination Enhances Fruit Quality and Economic Value. Agric. Ecosyst. Environ..

[124] Majewski J. (2016). Problem Wyceny Zapylania Jako Usługi Środowiskowej/The Problem of the Valuation of Pollination as Environment Service. Pr. Nauk. Uniw. Ekon. Wrocławiu.

[125] Kulhanek K., Bogaert J., Fauvel A.M., Hopkins B., Bogaert T. (2026). Enhanced Honey Bee Colony Strength and Economic Returns from Fall and Winter Feeding with a Complete Pollen-Replacing Feed. Insects.

[126] Feketéné Ferenczi A., Szűcs I., Bauerné Gáthy A. (2023). Evaluation of the Pollination Ecosystem Service of the Honey Bee (*Apis mellifera*) Based on a Beekeeping Model in Hungary. Sustainability.

[127] Abou Nader E., Kleftodimos G., Kyrgiakos L.S., Kleisiari C., Gallai N., Darwich S., Berchoux T., Vlontzos G., Belhouchette H. (2023). Linking Beekeepers’ and Farmers’ Preferences towards Pollination Services in Greek Kiwi Systems. Animals.

